# Zika virus infection and microcephaly: spatial analysis and socio-environmental determinants in a region of high *Aedes aegypti* infestation in the Central-West Region of Brazil

**DOI:** 10.1186/s12879-021-06805-1

**Published:** 2021-10-27

**Authors:** Patrícia Silva Nunes, Rafael Alves Guimarães, Celina Maria Turchi Martelli, Wayner Vieira de Souza, Marília Dalva Turchi

**Affiliations:** 1 Federal Institute of Education, Science and Technology of Goiás, Goiânia, Brazil; 2 Institute of Tropical Pathology and Public Health, Federal University of Goiás, Goiânia, Brazil; 3 Faculty of Nursing, Federal University of Goiás, Goiânia, Brazil; 4 Instituto Aggeu Magalhães, Oswaldo Cruz Foundation, Recife, Brazil

**Keywords:** Zika virus, Arbovirus, Socioeconomic status, Microcephaly, Public health, Spatial analysis, Brazil

## Abstract

**Background:**

More than 5 years after the Zika virus (ZIKV) epidemic, Zika infection remains a major concern in regions with high *Aedes* infestation. The objectives of this study were (i) to identify clusters of ZIKV infection and microcephaly, and/or central nervous system (CNS) alterations associated with congenital infection during the epidemic peak in 2016 and subsequently, in 2017 and 2018; (ii) to measure the non-spatial correlation between ZIKV infection and microcephaly and/or CNS alterations associated with congenital infection; and (iii) to analyse the sociodemographic/economic, health, and environmental determinants associated with the incidence of ZIKV in a region of high infestation by *Aedes aegypti* in the Central-West Region of Brazil.

**Methods:**

This ecological study analysed 246 municipalities in the state of Goiás (6.9 million inhabitants). The data were obtained from the Information System for Notifiable Diseases (ZIKV cases) and the Public Health Event Registry (microcephaly and/or CNS alterations associated with congenital infection). Incidence rates and prevalence of ZIKA infection were smoothed by an empirical Bayesian estimator (LEbayes), producing the local empirical Bayesian rate (LEBR). In the spatial analysis, ZIKV infection and microcephaly cases were georeferenced by the municipality of residence for 2016 and grouped for 2017 and 2018. Global Moran's I and the Hot Spot Analysis tool (Getis-Ord Gi* statistics) were used to analyse the spatial autocorrelation and clusters of ZIKV infection and microcephaly, respectively. A generalised linear model from the Poisson family was used to assess the association between ecological determinants and the smoothing incidence rate of ZIKV infection.

**Results:**

A total of 9892 cases of acute ZIKV infection and 121 cases of microcephaly were confirmed. The mean LEBR of the ZIKV infection in the 246 municipalities was 22.3 cases/100,000 inhabitants in 2016, and 10.3 cases/100,000 inhabitants in 2017 and 2018. The LEBR of the prevalence rate of microcephaly and/or CNS alterations associated with congenital infection was 7 cases/10,000 live births in 2016 and 2 cases/10,000 live births during 2017–2018. Hotspots of ZIKV infection and microcephaly cases were identified in the capital and neighbouring municipalities in 2016, with new clusters in the following years. In a multiple regression Poisson analysis, ZIKV infection was associated with higher population density, the incidence of dengue, *Aedes* larvae infestation index, and average rainfall. The important determinant of ZIKV infection incidence reduction was the increase in households attended by endemic disease control agents.

**Conclusions:**

Our analyses were able to capture, in a more granular way, aspects that make it possible to inform public managers of the sentinel areas identified in the post-epidemic hotspots.

**Supplementary Information:**

The online version contains supplementary material available at 10.1186/s12879-021-06805-1.

## Background

The Zika virus (ZIKV) is an arbovirus whose main vector of transmission in urban areas is the *Aedes* mosquito. The ZIKV was isolated for the first time in the 1940s and is considered to be a zoonotic pathogen associated with rare cases of benign acute febrile disease. This virus has circulated for more than 5 decades, silently, and is restricted to some regions of Africa and Asia [1]. The ZIKV has attracted worldwide attention since 2007, after the outbreaks of exanthematic dengue-like disease in Micronesia and then in French Polynesia, followed by an outbreak of Guillain–Barre syndrome [2, 3]. The first autochthonous cases of ZIKV infection in the Americas were identified in the Northeast region of Brazil in early 2015 [4, 5]. The virus had spread throughout the Brazilian territory, with an increase in the number of Guillain–Barre syndrome cases and an unprecedented increase in the history of medicine in cases of congenital microcephaly [6]. Robust scientific evidence has confirmed the link between prenatal ZIKV infection and congenital Zika syndrome (CZS), of which microcephaly is only one feature [7–10].

It is estimated that 2.2 billion people live in areas at risk for ZIKV infection [11, 12]. Globally, tropical and subtropical regions are ripe for the autochthonous transmission of ZIKV, because of the combination of climatic factors, level of vector infestation, and socio-demographic conditions [13–15]. In 2019, 87 countries recorded ZIKV vector transmission, and 61 other countries had evidence of the presence of competent vectors but without reported cases of infection. Central and South America, and the Caribbean accumulate most cases of ZIKV infection and CZS [1, 16]. In these regions, the peak of the ZIKV epidemic was in 2015 and 2016, with a decrease during subsequent years [1]. Considering the existence of the ZIKV-naive population living in risk areas, there is a potential for new outbreaks in previously affected regions or expansion to other regions, such as in Angola and India, in 2018 [16].

Brazil was the epicentre of the ZIKV infection and microcephaly epidemics, which were most concentrated in the Northeast and Southeast regions of the country, on the Atlantic coast. Since then, ZIKV has spread throughout the country, with more than 19,000 suspected cases of CZS and approximately 3500 confirmed cases in 2020 [17]. The epidemiological pattern of explosive outbreaks (2015 and 2016), followed by an abrupt reduction in cases of acute disease, suggests the acquisition of protective immunity in the population [18]. In general, explosive epidemics followed by apparent epidemiological silence have been recorded in descriptive studies and modelling for different arboviruses that share the same vector, such as ZIKV, dengue virus, and Chikungunya virus [19–22].

Brazilian studies show a heterogeneous geographical distribution of ZIKV infection and microcephaly [23–26], potentially explained by climatic factors, vector abundance, and unfavourable socioeconomic conditions [27–29]. Epidemiological studies have mostly been conducted in areas with higher incidences of acute ZIKV disease and of microcephaly [27, 28, 30]. However, there is little known about the regions that were less affected in the first wave, such as the Central-West region. These areas, otherwise considered cold in national assessments [26], are potential risk areas for new outbreaks due to the susceptible population and favourable conditions for arboviruses. The objectives of the present study were as follows: (i) to identify clusters of ZIKV infection and microcephaly and/or central nervous system (CNS) alterations associated with congenital infection during the epidemic peak (2016) and in the subsequent years of 2017 and 2018; (ii) to measure the non-spatial correlation between ZIKV infection and microcephaly and/or CNS alterations associated with congenital infection; and (iii) to analyse sociodemographic/economic, health, and environmental determinants associated with the incidence of ZIKV in a region of high infestation by *Aedes aegypti* in the Central-West region of Brazil.

## Methods

### Study design and setting

**Fig. 1:**
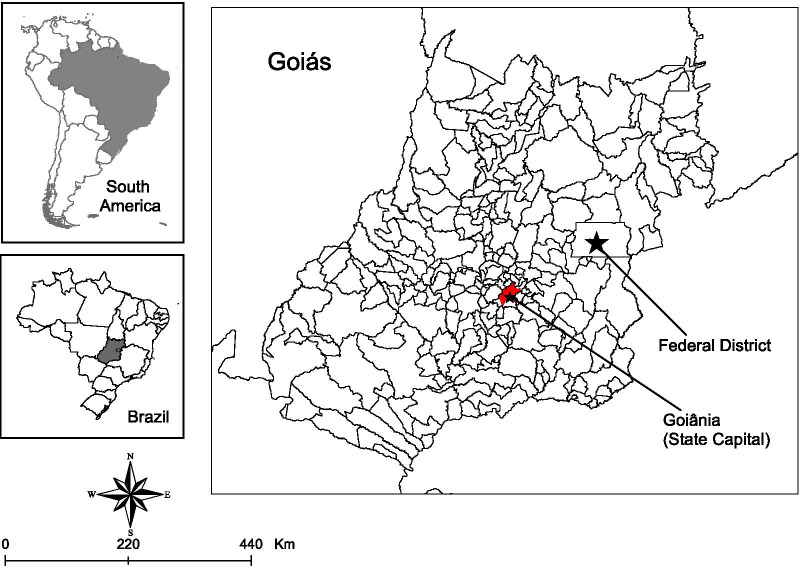
Study setting (Goiás, Central-West region, Brazil). *Note* State-level scale

An ecological study set in the state of Goiás was conducted from January 2016 to December 2018. Goiás is located in the Central-West region of Brazil and is made up of 246 municipalities in a 340,242.854 km^2^ area [31]. In 2020, its population was estimated at 7,113.540 million, with 4 million people living in the 14 municipalities with over 100,000 inhabitants [31]. The capital of Goiás is Goiânia. The capital’s metropolitan region encompasses 2.6 million inhabitants and 20 municipalities [31]. The municipalities of Brazil (the towns) are considered political and administrative divisions of the 26 Brazilian states and the Federal District [32]. The Federal District is an autonomous territory, without municipalities, located in Goiás with an independent structure (area: 5760.784 km^2^; population: 3055.149 million inhabitants) [31]. The Federal District, although located within Goiás, was not included in the analysis of the present study. Figure 1 shows the location of the study.

### Population

The study population comprised cases of confirmed ZIKV infection that occurred between 2016 and 2018 and were reported in the Information System for Notifiable Diseases (SINAN). Cases were confirmed by laboratory or clinical-epidemiological criteria, as recommended by the Ministry of Health of Brazil [33]. Cases confirmed by laboratory criteria were those that involved individuals with clinical suspicion of infection and who tested positive in one of the following laboratory tests: viral isolation, detection of viral ribonucleic acid, by reverse transcriptase polymerase chain reaction, and IgM serology for antibodies. In Brazil, after laboratory confirmation of the first cases of ZIKV infection in one area, the other acute cases of the disease are confirmed by clinical-epidemiological criteria, except for cases that involve pregnant women, children, patients with neurological manifestations, the elderly, and death, which should preferably occur by laboratory criteria. Cases confirmed by clinical-epidemiological criteria are those of individuals with clinical symptoms, which, in the impossibility of carrying out specific laboratory tests or inconclusive results, present an epidemiological link with a laboratory-confirmed case, after evaluation of the special distribution of confirmed cases [33].

The study also included confirmed cases of microcephaly and/or CNS alterations associated with congenital infection, reported in the Public Health Events Registry (RESP) [34], by municipality, in the same period. In Brazil, since the public health emergency of microcephaly and other alterations caused by ZIKV infection, several definitions of microcephaly in live births were adopted before the current standardised definition [34]. On 17 November 2015, microcephaly was defined as a condition where the head circumference (HC) is equal to or less than 33 cm for both sexes, when considering term children (37 or more weeks of gestation) as a reference [35]. The reference measure of HC was reduced to 32 cm or less for full-term children of both sexes in December 2015 [36]. In March 2016, the international standard definition of the World Health Organization (WHO) was adopted in Brazil, defining microcephaly as a HC equal to or less than 31.5 cm for term girls and equal to or less than 31.9 cm for term boys [37]. In August 2016, the new WHO definition based on intergrowth parameters was adopted, in which cases of microcephaly were considered as those born alive with HC lower than the cut-off point of -2 standard deviations below the mean value of the Fenton growth curves established according to sex and gestational age [38]. In addition to microcephaly, cases of other CNS alterations suggestive of congenital infection were included, including cerebral calcifications, hydrocephalus, schizencephaly/porencephaly, brainstem hypoplasia, ventriculomegaly, and other conditions [34]. In the present study, all cases of microcephaly and/or CNS alterations suggestive of congenital infection were accompanied by a consistent imaging diagnosis indicating an infectious cause and/or clinical-epidemiological or laboratory diagnosis of ZIKV or STORCH (infectious agents known to cause congenital infections, including syphilis, toxoplasmosis, cytomegalovirus, and herpes simplex virus) [34]. However, we found that at the time of exporting the database, of the total number of cases included in the study (n = 121), 65 (53.7%) were confirmed to be associated with the ZIKV [39].

The units of analysis in the study were all municipalities of Goiás. All cases of ZIKV infection and microcephaly and/or CNS alterations associated with congenital infection were analysed and georeferenced by the municipality of residence [17].

### Data sources and variables

The data used in this study were obtained on 28 February 2019. As indicated above, the study used secondary data from the SINAN (cases of ZIKV infection) and RESP (microcephaly and/or CNS alterations associated with congenital infection).

The SINAN includes data on diseases and conditions that are on the Ministry of Health’s list of diseases requiring compulsory notification, including suspected or confirmed cases of ZIKV infection, that must be updated weekly by all health units, public or private, in Brazil [40]. The RESP is an online form developed for recording public health emergencies, aiming at urgent surveillance actions. Since December 2015, the notification of microcephaly and/or alterations in the CNS, manifested in utero, birth, or childbirth, using this form, is compulsory, on a continuous basis, for all health units in Brazil [41].

Additionally, data on the total resident population of each municipality were obtained from estimates of the Brazilian Institute of Geography and Statistics [42], and the number of live births by year was obtained from the Information System on Live Births of the Ministry of Health of Brazil [43].

The crude ZIKV infection rate was calculated for each municipality by the number of confirmed cases divided by the population at risk (total resident population) multiplied by 100,000 [17]. This indicator was used to obtain smoothed rates, which, in turn, was used as a dependent variable.

The crude prevalence rate of microcephaly and/or CNS alterations associated with congenital infection was calculated for each municipality by the number of confirmed cases divided by the number of live births multiplied by 10,000 [23].

Three sets of indicators were used as independent variables to analyse the ecological determinants associated with the incidence of ZIKV infection: (i) sociodemographic/economic (per capita family income, gross domestic product per capita, municipal human development index, Gini index, and population density); (ii) health indicators, defined as synthesis measures that contain relevant information on certain attributes and dimensions of health status, as well as the performance of the health system [44] (dengue fever incidence rate, chikungunya incidence rate, population coverage of community health agents, and population coverage of endemic disease control agents); and (iii) environmental indicators (proportion of the population without access to sewage collection and treatment, proportion of the population living in households with piped water, proportion of the population living near a landfill site, average rainfall, and building infestation index for *Aedes* larvae). Additional file 1: Appendix S1 presents the reference year(s), data type, definitions, unity, and data sources of the independent variables.

### Statistical analysis and modelling

Data analysis was performed in five steps.

#### Smoothing rate

This step consisted of smoothing the ZIKV infection rate and crude prevalence rate of microcephaly and/or CNS alterations associated with congenital infection.

Due to the small number of cases and/or the total resident population of many municipalities in Goiás, the crude ZIKV infection rate was smoothed using a Bayesian model [45]. For smoothing, we used the Empirical Bayes method to estimate the spatial empirical Bayesian rate or local empirical Bayesian rate (LEBR) through a local empirical Bayesian estimator (LEbayes). Briefly, an empirical Bayesian rate or global empirical Bayesian rate of a region is the weighted sum of its crude rate and the global mean rate. The global empirical Bayesian rate can be further improved in the case of small populations by including spatial neighbourhood effects in their estimates. For this, the LEBR, whose weighting is made by the local mean value, uses the mean of the geographic neighbourhood close to the area in which the rate is to be estimated, instead of the global mean rate [45–47]. This method allowed the smoothing of random fluctuations in the crude rate of ZIKV infection in the municipalities. Similarly, we also calculated the LEBR of the prevalence rate of microcephaly and/or CNS alterations associated with congenital infection. Smoothing was carried out using TerraView software, version 5.6.1 [48].

#### Descriptive analysis

A monthly descriptive analysis of the number of cases of ZIKV infection and microcephaly and/or CNS alterations associated with congenital infection between January 2016 and December 2018 was performed. A descriptive analysis, using the average of the 246 municipalities, of these indicators for each year (2016, 2017, and 2018) and for the post-epidemic period (2017–2018) was also performed.

#### Spatial analysis

Initially, to verify the existence of spatial autocorrelation of the LEBR of ZIKV infection and cases of microcephaly and/or CNS alterations associated with congenital infection, Moran's I index was used. The values of Moran's I vary from − 1 (maximum negative association) to + 1 (maximum positive association), and the Z score and p-value were used to analyse the statistical significance of Moran's I [49].

A spatial cluster analysis was then performed to identify clusters of the LEBR of ZIKV infection and cases of microcephaly and/or CNS alterations associated with congenital infection, separately. In this analysis, the Hot Spot Analysis tool (Getis-Ord Gi*) [50] was used, which identifies two types of clusters: hotspots, which are areas with high incidence of diseases, and coldspots, which are areas of low incidence [51]. Briefly, the Getis-Ord Gi* is estimated by comparing the local sum for a feature and its neighbours with the sum of all features [52]. The Z-score was used to verify significant hotspots/coldspots; when the local sum is very different from the expected local sum, and that difference is too large to be the result of random chance, a statistically significant Z-score is obtained [52–54]. According to the Z-scores, the municipalities were classified as hot or cold areas at a significance level of 90% (p-value < 0.10), 95% (p-value < 0.05) or 99% (p-value < 0.01) [55]. The conceptualisation of spatial relationships was based on fixed distances [52]. The year 2016 (epidemic year) and the period of 2017–2018 (post-epidemic years) were considered in this analysis.

All spatial analyses related to ZIKV infection used LEBR, that is, smoothed rates. Despite the calculation of the smoothed prevalence rate of microcephaly and/or CNS associated with congenital analysis, we used the number of microcephaly cases for cluster analysis, because even after smoothing, cities with many zeros and/or low rates were obtained.

The Moran's I index and Getis-Ord Gi* geospatial analysis, were performed using ArcGIS software, version 10.3 [55].

#### Non-spatial correlation

The Spearman correlation coefficient (r_s_) was used to analyse the correlation between the mean LEBR of ZIKV infection (2016–2018) and the sum of the number of microcephaly cases of microcephaly and/or CNS alterations associated with congenital infection (2016–2018). Statistical significance was set at p-values < 0.05.

#### Determinants associated with ZIKV infection

The dependent variable in the study was the mean LEBR of ZIKV infection between 2016 and 2018, which was determined by analysing 246 observations (246 municipalities of the Goiás). The independent variables were the sociodemographic/economic, health, and environmental indicators of the 246 municipalities described in Additional file 1: Appendix S1.

To analyse the association between the dependent and independent variables, we used a sequential generalised linear model from the Poisson family [56].

In the bivariate Poisson analysis, the magnitude and significance of the associations between each of the independent variables and the dependent variable were initially verified [57]. The results of the bivariate analysis are presented as the crude incidence rate ratio, 95% confidence interval, regression coefficient, standard error, and p-value.

Variables that presented p-value < 0.20 in the bivariate analysis were considered for inclusion in the model. We first used the Spearman correlation matrix and the variance inflation factor (VIF) to detect multicollinearity between the independent variables [58, 59]. To avoid potential collinearity, we excluded variables with a correlation coefficient equal to or higher than 0.6 (r_s_ > 0.6) or VIF ≥ 10.0 [59]. After excluding potentially collinear variables, three Poisson regression models were constructed in a hierarchical structure. In model 1, sociodemographic/economic variables were included; in model 2, the variables of health indicators (+ sociodemographic/economic variables); and in model 3 (final model) the environmental variables (+ sociodemographic/economic variables + health variables). The Akaike information criterion was used to compare the three regression models. The results of the multiple regression analysis are presented as adjusted incidence rate ratio, 95% confidence interval, regression coefficient, standard error, and p-value. Wald's test was used to analyse statistical significance [60]. Statistical significance was set at p-value < 0.05.

Poisson's analysis was conducted in the STATA statistical program, version 15.0 (StataCorp, College Station, TX, USA).

## Results

### Descriptive analysis

Between January 2016 and December 2018, 18,495 suspected cases of ZIKV infection were reported in the state of Goiás. Of these, 9892 (53.5%) were confirmed; 9005 (91.0%) by clinical-epidemiological criteria and 887 (9.0%) by laboratory criteria. Of these, 69.9% (n = 6913) were women.

**Fig. 2:**
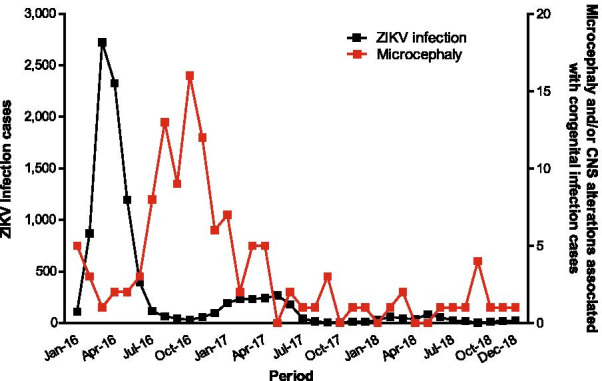
Monthly distribution of ZIKV infection and microcephaly and/or CNS alterations associated with congenital infection cases in Goiás, Central-West region of Brazil, 2016–2018. *Note* State-level scale

Figure 2 shows the absolute monthly number of cases of ZIKV infection and microcephaly and/or CNS alterations associated with congenital infection in Goiás between January 2016 and December 2018. The number of cases for each year were as follows: 2016 (n = 8026), 2017 (n = 1448), and 2018 (n = 418). The highest absolute number of cases of ZIKV infection (n = 7620) occurred in the first half of 2016, rising again in the same period of the following year, with 1349 cases. There were 121 cases of microcephaly and/or CNS alterations associated with congenital infection: 81 in 2016, 26 in 2017, and 14 in 2018. The greatest number of cases occurred in the second half of 2016, with 64 records.

The mean crude incidence rate of ZIKV infection in the 246 municipalities of Goiás was 16.9, 13.2, and 3.4 cases/100,000 inhabitants in 2016, 2017, and 2018, respectively. In the period of 2017–2018 (post-epidemic years), the mean incidence rate was 8.3 cases/100,000 inhabitants, which was lower than that in 2016. The mean LEBR of ZIKV infection in the municipalities was 22.3, 16.3, and 5.5 cases/100,000 inhabitants, in 2016, 2017, and 2018, respectively. In 2017–2018, the mean LEBR was 10.3 cases/100,000 inhabitants, which was also lower than that in 2016.

The mean crude prevalence rates of microcephaly and/or CNS alterations associated with congenital infection were 5, 3, and 1 cases/10,000 live births, respectively. The crude mean was 2 in the 2017–2018 period, which was lower than that in 2016 (epidemic year). The mean LEBR prevalence rates of microcephaly and/or CNS alterations associated with congenital infection were 7, 3, and 1 cases/10,000 live births, respectively. In 2017–2018, the mean LEBR was 2 cases/10,000 live births, which was lower than that in 2016.

### Spatial analysis

Moran’s I global indexes for the LEBR of ZIKV infection were 0.068 (p-value = 0.002) and − 0.007 (p-value = 0.908) in 2016 and 2017–2018, respectively. These results suggest the presence of spatial autocorrelation of the LEBR of ZIKV infection in 2016, but not during 2017–2018.

**Fig. 3:**
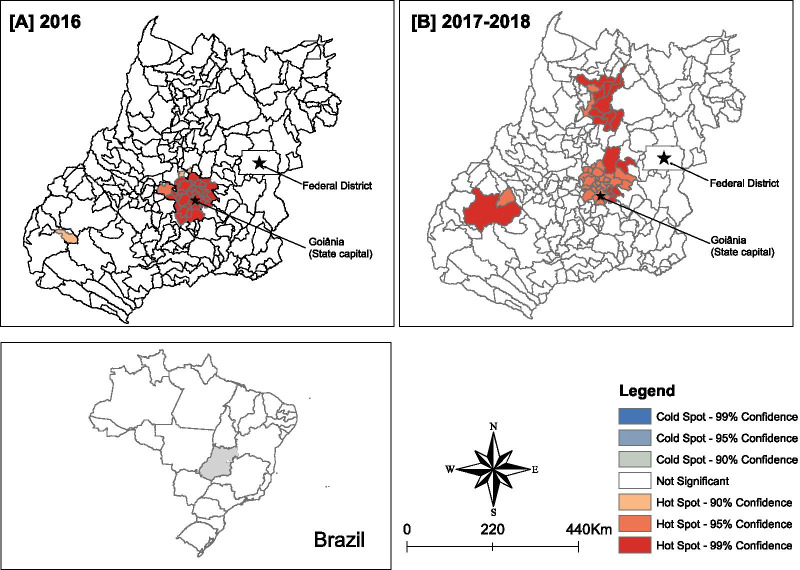
Spatial clusters (Getis-Ord Gi*) of the LEBR of ZIKV infection in Goiás, Central Brazil, 2016 and 2017–2018. *Note* State-level scale

Additional file 2: Figure S1 presents the results of the descriptive analysis of the LEBR of ZIKV infection in Goiás. Figure 3 presents the results of the spatial cluster analyses (Getis-Ord Gi*) for the LEBR of ZIKV infection in 2016 and 2017–2018.

In 2016, we identified a cluster with a high incidence of ZIKV infection, with a significance greater than or equal to 90%. The cluster included 34 municipalities in the metropolitan region of the capital and surrounding municipalities (population of 2.9 million). Of these, 32 municipalities formed a cluster with 99% significance. We identified a hotspot with a 90% significance level, composed of a single municipality (with a population of 3,100 inhabitants), isolated from the initial cluster and located in the southwest region of the state. No coldspots were identified (Fig. 3A). For the period of 2017–2018, we identified two other clusters with a high incidence of ZIKV infection, with significance equal to or higher than 90%, located in the north (10 municipalities with 108,000 inhabitants) and southwest (two municipalities with 22,000 inhabitants). No coldspots were identified (Fig. 3B).

Cases of microcephaly and/or CNS alterations associated with congenital infection were distributed in 28, 14, and 9 municipalities in 2016, 2017, and 2018, respectively. The Moran’s I global indexes were 0.072 (p-value < 0.001) and 0.135 (p-value < 0.001) in 2016 and 2017–2018, respectively, suggesting the presence of spatial autocorrelation in both periods of analysis.

Additional file 3: Figure S2 presents the descriptive analysis of the rate of prevalence of microcephaly and/or CNS alterations associated with congenital infection in Goiás, and Additional file 4: Figure S3 presents the municipalities with confirmed cases.

**Fig. 4:**
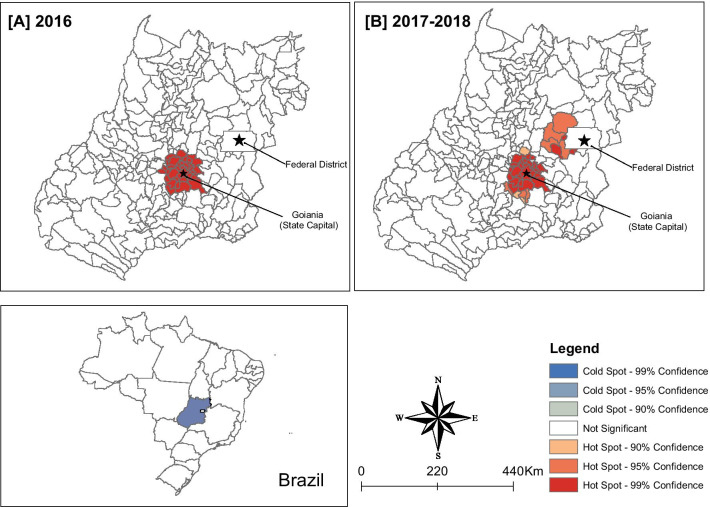
Getis-Ord Gi* spatial clusters of microcephaly cases in Goiás, Central-West region of Brazil, 2016 and 2017–2018. *Note* State-level scale

Figure 4 presents the spatial cluster analysis (Getis-Ord Gi*) of the 121 cases of microcephaly in Goiás in 2016 and 2017–2018. For 2016, a cluster with 99% significance was identified, encompassing 33 municipalities in the capital's metropolitan region and surrounding municipalities (population of 2.9 million inhabitants) (Fig. 4A). For 2017–2018, we identified a second spatial cluster with a significance of 95% or more, encompassing eight municipalities with approximately 627,000 inhabitants, located around the Federal District. No coldspots were identified (Fig. 4B).

### Non-spatial correlation analysis

Spearman's correlation analysis showed a moderate positive correlation between the LEBR of ZIKV infection and the sum of the number of microcephaly cases between 2016–2018 (r_s_ = 0.373; 95% CI 0.261–0.475; p < 0.001), indicating that in Goiás, the increase in the ZIKV infection incidence rate was associated with an increase in microcephaly cases.

### Determinants associated with ZIKV infection

#### Bivariate analysis

The bivariate analysis showed a statistically significant association between the mean incidence rate of ZIKV infection and all independent variables (p-value < 0.05), except for the chikungunya incidence rate (p-value = 0.142) (Additional file 5: Table S1).

#### Multivariable analysis

Before formulating the models, tests for collinearity were performed. The Spearman's correlation matrix showed a high correlation between per capita family income and municipal human development index (r_s_ = 0.787), indicating potential collinearity. The average VIF was 1.89, ranging from 1.03 for population coverage of endemic disease control to 5.68 for the per capita family income. We chose to remove per capita family, instead of the municipal human development index, since the latter also reflects the dimension of family income.

Model 1 included the sociodemographic/economic variables; Model 2 included, in addition to these variables and the health variables and, Model 3 (the final model) included the sociodemographic/economic variables, health variables, and the environmental variables. Model 3 showed the highest fit, lowest AIC value (6899.3), and highest R^2^ (0.315), compared with models 1 and 2. This model showed a positive association between the mean incidence rate of ZIKV infection and population density, incidence rate of dengue fever, average rainfall, and building infestation index for *Aedes* larvae, and a negative association with population coverage of endemic disease control agents (Table 1).

**Table 1:** Association between ecological determinants and zika virus infection in Goiás, Central-West region of Brazil, 2016–2018: multivariable analysis

Variables	Model 1				Model 2				Model 3			
	aIRR (95% CI)	β	SE	p-value	aIRR (95% CI)	β	SE	p-value	aIRR (95% CI)	β	SE	p-value
Sociodemographic/economic												
Gross domestic product per capita	1.06 (0.93–1.20)	0.058	0.070	0.376	0.98 (0.85–1.14)	− 0.018	0.073	0.811	0.92 (0.77–1.10)	− 0.083	0.082	0.350
Municipal human development index	1.98 (0.60–6.48)	0.684	1.199	0.257	1.19 (0.30–4.77)	0.175	0.844	0.805	0.59 (0.11–2.99)	− 0.522	0.490	0.527
Gini index	0.85 (0.38–1.92)	− 0.157	0.353	0.703	0.98 (0.48–2.02)	− 0.016	0.362	0.811	1.24 (0.53–2.88)	0.216	0.533	0.615
Population density	1.08 (0.99–1.17)	0.044	0.004	0.073	1.06 (0.98–1.14)	0.054	0.041	0.966	1.09 (1.07–1.10)	0.082	0.049	< 0.001
Health												
Dengue fever incidence rate					1.04 (1.01–1.06)	0.036	0.010	0.001	1.03 (1.01–1.06)	0.033	0.045	0.003
Chikungunya incidence rate					0.89 (0.48–1.62)	− 0.120	0.274	0.696	0.99 (0.56–1.75)	− 0.010	0.288	0.970
Population coverage of community health agents					0.99 (0.84–1.18)	− 0.004	0.087	0.962	1.05 (0.88–1.26)	0.051	0.098	0.578
Population coverage of endemic disease control agents					0.80 (0.61–1.05)	− 0.221	0.109	0.102	0.74 (0.57–0.96)	− 0.052	0.098	0.023
Environmental												
Proportion of the population without access to sewage collection and treatment									0.95 (0.87–1.04)	0.533	0.042	0.241
Proportion of the population living in a household with piped water									1.70 (0.83–3.52)	0.427	0.630	0.149
Proportion of the population living in a garbage collection site									1.53 (0.46–5.06)	0.633	0.935	0.483
Average rainfall									1.88 (1.35–2.61)	0.348	0.313	< 0.001
Building infestation index for *Aedes* larvae									1.42 (1.32–1.52)	0.345	0.050	< 0.001
AIC	9433.6				7752.2				6889.3			
R^2^	0.063				0.229				0.315			

Model 1: sociodemographic/economic variables

Model 2: sociodemographic/economic variables + health variables

Model 3: Sociodemographic/economic variables + health and environmental variables

*AIC* Akaike information criterion; *β* regression coefficient; *95% CI* 95% confidence interval; *aIRR* adjusted incidence rate ratio; *SE* standard error; *R*^2^ determination coefficient; *Wald test

## Discussion

Our results showed that the first outbreak of ZIKV infection, followed by an increase in the cases of microcephaly and/or CNS alterations associated with congenital infection occurred in 2016 in Goiás, in the Central-West region of Brazil. In this year of introduction of the ZIKV in the interior of the country, there was a clear presence of clusters of both diseases in the capital and the neighbouring municipalities, which presented the highest population density in the state. In the following years (2017–2018), there was an approximate four-fold reduction in the incidence of ZIKV infection, along with the expansion of risk areas with the identification of new clusters located farther from the initial hotspot. In multiple regression analysis, ZIKV infection was positively associated with higher population density in the municipality, incidence of dengue fever, *Aedes* larvae infestation index, and average rainfall. The important determinant of ZIKV infection incidence rate reduction was the increase in population coverage of endemic disease control agents.

In our study, the peak of ZIKV infection in Goiás was detected in the first half of 2016, less than a year after the first wave of infection in the Northeast region of Brazil [23]. Therefore, there was a rapid spread of ZIKV infection due to population mobility [26], high infestation of *Aedes aegypti*, and regional climate characteristics [61–63], or by the presence of a population totally or partially susceptible to ZIKV infection [1]. The combination of high temperatures and high rainfall rates that favour the proliferation of vectors is a factor associated with ZIKV infection in Colombia [64]. The peak of microcephaly cases in Goiás occurred approximately 6 months after the outbreak of ZIKV infection. This epidemiological pattern, also described for other regions of Brazil [23, 65], is compatible with the biological plausibility of the adverse outcomes of ZIKV infection in pregnancy [7, 29]. Before 2015, the estimated prevalence of microcephaly was 2 per 10,000 live births, which was stable in the years 2000–2014 in Brazil [23, 66]. In 2016, the maximum prevalence of microcephaly was 56.7 per 10,000 live births in the Northeast region, with intermediate risks (14.4 per 10,000 live births) estimated for the Central-West region [23], along with an evident disparity in the magnitude of microcephaly outbreaks between municipalities [26].

In our study, the persistence of ZIKV infection clusters/hotspots in and around the metropolitan region during the outbreak and in subsequent years can be explained, at least partially, by the high population density of the region. This correlation between high population density and an increase in ZIKV infection incidence rate is in agreement with the spatial analysis of data obtained from different states of the Northeast region [21] and at the national level [23, 26]. One study that examined countries in the Americas added to the evidence suggesting that population density and the level of infestation are risk factors for transmission of ZIKV infection between countries [67]. In the present study, spatial analysis also identified new clusters of ZIKV infection in the post-outbreak years. These new clusters encompassed municipalities that had lower population density and were distant from the initial epicentre, were located in the north and southwest regions of the state, and were centres of agricultural activity and ecotourism (North – Serra da Mesa; West – Parque Nacional das Emas). These findings suggest that there was a rapid dispersion of ZIKV, with an expansion of the ZIKV transmission risk area in the years 2017–2018, and that even municipalities with lower population densities were affected.

The 2016 epidemic hotspots for both ZIKV infection and microcephaly and/or CNS alterations associated with congenital infection were detected in the state capital and surrounding municipalities. The post-epidemic (2017–2018) spatial analysis detected a new cluster of cases, approximately 200 km from the capital, in municipalities surrounding the Federal District. It is interesting to note that this new cluster did not overlap with the hotspots identified for ZIKV infections during the same period. This apparent discrepancy may, in our opinion, be explained by the low clinical impact of the ZIKV cases and/or by the potential possibility of the cases utilising health services in the federal capital, and the possible overlap of surveillance data between the bordering regions. It is noteworthy that the detection of this microcephaly cluster and/or alterations in the CNS associated with congenital infection may have occurred as a result of the small number of cases in the region. Further field investigations are required to verify the presence of a true cluster in the metropolitan region. The hotspot for microcephaly and/or CNS alterations associated with congenital infection cases identified in this study (2017–2018) were not detected in the national-level kernel analysis that also used the RESP database [23]. Consistent with our study findings, a spatial–temporal analysis of the microcephaly outbreak in Pernambuco state (2015), in the Northeast region of Brazil, also showed a high concentration of cases in the metropolitan area of the capital, followed by the expansion of microcephaly cases to municipalities with smaller populations, far from the initial epicentre [68]. The present spatial analysis contributes to existing research by identifying other areas of risk for the occurrence of microcephaly, in addition to the metropolitan areas of the capital and its surroundings. These hotspots, along with the capital and surrounding areas, should be considered as monitoring sentinels for cases of ZIKV infection and microcephaly and/or CNS alterations associated with congenital infection.

In line with the results of the spatial analysis, the multivariable analysis showed that higher ZIKV infection incidence rates were associated with municipalities with a higher population density. Our study showed that for every 10% increase in the coverage of municipalities by endemic disease control agents, there was a reduction of approximately 26% in the incidence of ZIKV infection, regardless of other explanatory factors included in the model. In Brazil, the endemic disease control agents are professionals who are part of the health surveillance team and carry out vector surveillance activities, such as surveys of homes, vacant lots, warehouses, and commercial establishments. Gutters, roofs, and water tanks are also inspected. These professionals are closely linked to vector control and the health education of the population. They contribute substantially to the elimination of vectors and, similarly, to the control of arboviruses, including Zika [69]; therefore, they play a fundamental role in the reduction of the magnitude of ZIKV infection.

Other predictive factors of an increased ZIKV infection rate were average rainfall and the building infestation index for *Aedes* larvae, which represents the percentage of housing properties showing the presence of *Aedes aegypti* larvae. This vector index reflects the presence of urban breeding sites, resulting from the disorderly growth of cities without adequate infrastructure for water supply, sanitation, and waste management services [27, 70, 71]. The incidence of dengue as a predictor of ZIKV infection is a proxy for the local transmission of arboviruses, also identified at the national level [29, 72]. The same was not observed for the incidence of Chikungunya, which can be explained by the low circulation of this virus in the studied region [17]; there is a higher concentration of cases in states in the Northeast region of the country. This finding can also be explained by the recentness of the introduction of the chikungunya virus in the Central-West region of Brazil in 2015, compared with the dengue virus that has been circulating in the region for at least 2 decades.

In the multiple model regression analysis carried out in the present study, sociodemographic/economic conditions at the municipality level were not found to be predictors of ZIKV infection. This finding contradicted that of a study set in the capital of Pernambuco that used the stratification of socioeconomic conditions by neighbourhoods [30]. The modelling used in that study, which included single-entry predictors in the final model, might have contributed to this finding. Additionally, the spatial analysis unit of the present study, which encompassed different social strata, might have made these indicators less discriminatory between municipalities. Other studies that analysed the Northeast and Southeast regions of Brazil found associations between the incidence of ZIKV and factors such as population density, human development index, primary care coverage, violence rate, average household income, and race, thereby reinforcing the association of the social determinants of health with the dispersion of ZIKV and the worsening of congenital cases [28, 67, 73].

The use of secondary data from passive reporting is an inherent limitation of this type of study, considering that epidemiological surveillance records probable cases of acute ZIKV disease via access to health services. Thus, the ability to detect cases may vary among municipalities, depending on the health network structure and surveillance. These epidemiological surveillance data may be underestimated, considering that ZIKV infection is mostly asymptomatic and clinical manifestations are generally mild, with rare neurological complications [1, 8]. In Brazil, access to health services is universal and free through the Unified Health System, and SINAN surveillance is compulsory nationwide [74]. In the present study, we analysed the spatial and temporal distribution of acute ZIKV diseases confirmed by laboratory and/or clinical-epidemiological criteria. We excluded inconclusive cases, thus minimising the risk of classification errors. Even so, there is still the possibility of diagnostic error due to the clinical similarity of ZIKV with other exanthematic arboviruses (such as the dengue, chikungunya, and Mayaro viruses) that circulate in the region [22, 75, 76]. The compulsory notification of ZIKV infection and microcephaly associated with the infection (RESP) in Brazil, although recent (February 2016), has been shown to be consistent in discriminating between outbreaks and inter-regional differences [23, 29, 74]. The criteria for defining the cases of microcephaly associated with congenital CZS in the RESP require, in addition to specialised clinical evaluation, laboratory tests and/or imaging, which makes it difficult to confirm the aetiology of the cases. However, the significant increase in microcephaly cases after the ZIKV epidemic reinforces the association between these two diseases and the consistency of the analysed data.

It is important to note that probable cases of ZIKV infection continue to be reported in 2019 and 2020 owing to the persistence of viral circulation in Brazilian regions [17]. This, in addition to the existence of a naive population, vector abundance, and favourable climatic conditions, alerts to the threat of new outbreaks of ZIKV and microcephaly and/or CNS alterations associated with congenital infection in the region studied.

## Conclusions

The results highlighted the importance of the role of endemic disease control agents and the need for improvement in vector control activities, considering that there is no vaccine or specific treatment for the acute disease and its neurological repercussions. In addition, factors such as population density, dengue fever incidence rate, average rainfall, and building infestation index for *Aedes* larvae should be monitored because of their association with an increase in the occurrence of ZIKV infection. Our spatial analysis and statistical model were able to capture, in a more granular way, aspects that make it possible to inform public managers of the sentinel areas identified in the post-epidemic hotspots, with the expansion of risk areas and the emergence of new clusters located far from the hotspot initially found during the epidemic period.

## Supplementary Information

**Additional file 1: Appendix 1. **Independent variables usedin the proposed model.

**Additional file 2: Figure S1. **Descriptive spatial analysis of local empirical Bayesian rate of ZIKV infection in Goiás,Central-West region of Brazil, 2016-2018. Note: State-level scale.

**Additional file 3: Figure S2. **Descriptive spatial analysis of local empirical Bayesian rate of microcephaly and/or central nervous system alterations associated with congenital infection in Goiás, Central-West region of Brazil, 2016-2018. Note:State-level scale.

**Additional file 4: Figure S3. **Municipalities with confirmed cases of microcephaly and/or central nervous system alterations associated with congenital infection in Goiás, Central-West region of Brazil,2016-2018. Note: State-level scale.

**Additional file 5: Table S1.** Association between ecological determinants and the mean incidence rate of Zika virus infection inGoiás, Central-West region of Brazil, 2016-2018: bivariate analysis.

## Data Availability

The data used in this manuscript are found in repositories with public hyperlinks: https://datasus.saude.gov.br/trabalho-e-renda-censos-1991-2000-e-2010 (Demographic census 2010); http://www.atlasbrasil.org.br/ (Atlas of Human Development in Brazil); https://portalsinan.saude.gov.br (National System of Notifiable Diseases); https://egestorab.saude.gov.br/ (Department of Primary Care); http://cnes.datasus.gov.br/ (National Register of Health Establishments); https://www.snirh.gov.br/ (National Water and Basic Sanitation Agency); https://portal.inmet.gov.br/ (National Institute of Meteorology); https://extranet.saude.go.gov.br/sacd/EstatisticaQuadrasVisitadas.jsf (*Aedes* Zero Integrated Monitoring System).
