# Supplementary material for: Zika virus infection and microcephaly: spatial analysis and socio-environmental determinants in a region of high Aedes aegypti infestation in the Central-West Region of Brazil
Source: BMC Infect Dis. 2021 Oct 27;21:1107. doi: 10.1186/s12879-021-06805-1 (PMC8549329; doi:10.1186/s12879-021-06805-1)
Supplement: Supplementary file 1 — Additional file 1: Appendix 1. Independent variables usedin the proposed model. [file 12879_2021_6805_MOESM1_ESM.docx]

**Appendix 1.** Independent variables used in the proposed model.

| **Independent variables** | **Year** | **Type of data** | **Definition** | **Unit** | **Source** |
| --- | --- | --- | --- | --- | --- |
| **Sociodemographic/economic** | | | | | |
| Per capita family income | Annual/2010 | Mean/Continuous | Mean per capita household income of people residing in a given geographic area, in the year under consideration. Per capita household income was defined as the sum of monthly income of household residents, in R$, divided by the number of residents. | R$ 100.00 | Brazilian Institute of Geography and Statistics [1] |
| Gross domestic product per capita | Annual/2010 | Mean/Continuous | Average value added per individual, in currency and at market prices, of the final goods and services produced in a given geographic space, in the year under consideration. It measures the fraction of the GDP that each individual in a country would have if they all received equal shares. | R$ 10,000.00 | Brazilian Institute of Geography and Statistics  [1] |
| Municipal human development index | Annual/2010 | Index/Continuous | Geometric mean of the indices of the dimension’s income, education, and longevity with equal weights. Municipal Human Development Index - Education Dimension (2010): index obtained using the geometric mean of the subindex of attendance of children and young people to school, with a weight of 2/3, and the subindex of education of the adult population, with a weight of 1/3. Municipal Human Development Index - Longevity Dimension (2010): index obtained based on the life expectancy at birth using the formula: [(observed value of the indicator) - (minimum value)] / [(maximum value) - (minimum value)], considering as the minimum and maximum values ​25 and 85 years, respectively. Municipal Human Development Index - Income Dimension (2010): index obtained based on the per capita income indicator using the formula: [(observed value of the indicator) - (minimum value)] / [(maximum value) - (minimum value)], considering as the minimum and maximum values R$ 8.00 and R$ 4,033.00 (prices in August 2010). | 0.1 point | Atlas of Human Development in Brazil [2] |
| Gini index | Annual/2010 | Index/Continuous | It measures the degree of concentration of the distribution of household income per capita in a given population and in a given geographic space. Its value ranges from 0, when there is no inequality (the per capita household income of all individuals has the same value), to 1, when inequality is maximum (only one individual has all the income). | 0.1 point | Brazilian Institute of Geography and Statistics [1] |
| Population density | Annual/2010 | Ratio/Continuous | Demographic index that assesses the distribution of the population in a given territory. | 100 inhab/km² | Brazilian Institute of Geography and Statistics [1] |
| **Health** | | | | | |
| Dengue fever incidence rate | Annual/2016-2018 | Mean rate/Continuous | Number of new reported cases of dengue (classic and hemorrhagic fever) per 100,000 inhabitants, in the population residing in a given geographic area, in the year under consideration. The definition of a dengue case is based on criteria adopted by the Ministry of Health to guide the epidemiological surveillance of the disease across the country, and considers as a suspected case of dengue an individual residing in an area where there are cases of dengue or who has traveled in the last 14 days to an area with transmission or presence of *Aedes aegypti*, showing clinical signs of fever (between 2 and 7 days), and two or more of the following manifestations: nausea/vomiting; rash; myalgia/arthralgia; headache/retroorbital pain; petechiae/positive loop proof; leukopenia. | 50 cases/100,000 inhabitants | National System of Notifiable Diseases [3] |
| Chikungunya incidence rate | Annual/2016-2018 | Mean rate/Continuous | Number of new reported cases of chikungunya per 100,000 inhabitants, in the population residing in a given geographic area, in the year under consideration. The definition of a chikungunya case is based on criteria adopted by the Ministry of Health to guide the epidemiological surveillance of the disease across the country and considers as a suspected case of Chikungunya an individual with sudden onset fever higher than 38.5°C and severe pain in the joints (arthralgia) or severe arthritis of acute onset, not explained by other conditions, residing in or having visited areas where suspected cases are occurring up to two weeks before the onset of symptoms or that are linked to a confirmed case. | 50 cases/100,000 inhabitants | National System of Notifiable Diseases [3] |
| Population coverage of community health agents | Annual/2016-2018 | Mean proportion/Continuous | Population coverage estimated by community health workers linked to the Family Health Strategy teams, the parameterized Primary Care team, and the community health workers team, given by the percentage of the population covered by these agents in relation to the population estimate. | 10% | Department of Primary Care – [4] |
| Population coverage of endemic disease control agents | Annual/2016-2018 | Mean proportion/Continuous | Coverage of households by endemic disease control workers given by the percentage of properties in the territory covered by these agents, in relation to the total estimated properties in the area. | 10% | National Register of Health Establishments – [5] |
| **Environmental** | | | | | |
| Proportion of the population without access to sewage collection and treatment | Annual/2013 | Proportion/Continuous | Percentage of the resident population that does not have sewage disposal through the connection of the household to the sewage network, in a given geographical area, in the year under consideration. | 10% | National Water and Basic Sanitation Agency [6] |
| Proportion of the population living in a household with piped water | Annual/2010 | Proportion/Continuous | Percentage of the resident population served by the public water-supply system, in a given geographic area, in the year under consideration. The water can come from the general system, a well, a spring, or a reservoir supplied by rainwater or water tankers. | 10% | National Water and Basic Sanitation Agency [6] |
| Proportion of the population living in a garbage collection site | Annual/2010 | Proportion/Continuous | Percentage of the resident population served, directly or indirectly, by a regular household garbage collection service, in a given geographic area, in the year under consideration. This includes situations in which garbage collection is carried out directly by a public or private company, or the garbage is deposited in a garbage container, tank, or warehouse outside the home, for later collection by the service provider. Only permanent private households located in urban areas are considered. | 10% | Atlas of Human Development in Brazil [2] |
| Average rainfall | Monthly/2016-2018 | Mean/Continuous | The amount of rain that falls in a certain place and at a certain time is measured by the pluviometer and registered by the pluviograph. Precipitation is considered to be all forms of water, liquid or solid, that fall from clouds and reach the ground: drizzle, icy drizzle, cold rain, hail, ice crystals, ice balls, rain, snow, snowballs, and snow particles. Volume expressed in millimeters (mm) referring to the state of water - whether liquid or solid - that falls over a given region and for a given period. | 50 mm | National Institute of Meteorology – [7] |
| Building infestation index for *Aedes* larvae | Monthly/2016-2018 | Mean index/Continuous | Index that reflects the percentage of positive properties (with the presence of *Aedes aegypti* larvae). It is produced by analyzing the mosquito larvae collected in the properties by endemic disease control workers. | 1% | *Aedes* Zero Integrated Monitoring System [8] |

**References**

[1] Brazilian Institute of Geography and Statistics. Demographic census 2010. Available in: <https://datasus.saude.gov.br/trabalho-e-renda-censos-1991-2000-e-2010>. Accessed 02 June 2020.

[2] Atlas (BR). Atlas of Human Development in Brazil. Available in: <http://www.atlasbrasil.org.br/>. Accessed 02 June 2020.

[3] Ministry of Health (BR). National System of Notifiable Diseases (SINAN). Available in: <https://portalsinan.saude.gov.br>. Accessed 02 June 2020.

[4] Ministry of Health (BR). Department of Primary Care. Available in: <https://egestorab.saude.gov.br/>. Accessed 02 June 2020.

[5] Ministry of Health (BR). National Register of Health Establishments. Available in: <http://cnes.datasus.gov.br/>. Accessed 02 June 2020.

[6] Ministry of the Environment (BR). National Water and Basic Sanitation Agency. Available in: <https://www.snirh.gov.br/>. Accessed 02 June 2020.

[7] Ministry of Agriculture, Livestock and Supply (BR). National Institute of Meteorology. Available in: <https://portal.inmet.gov.br/>. Accessed 02 June 2020.

[8] State Department of Health of Goias. *Aedes* Zero Integrated Monitoring System. Available in: <https://extranet.saude.go.gov.br/sacd/EstatisticaQuadrasVisitadas.jsf>. Accessed 02 June 2020.
