# Supplementary figures and images for: Zika virus infection and microcephaly: spatial analysis and socio-environmental determinants in a region of high Aedes aegypti infestation in the Central-West Region of Brazil
Source: BMC Infect Dis. 2021 Oct 27;21:1107. doi: 10.1186/s12879-021-06805-1 (PMC8549329; doi:10.1186/s12879-021-06805-1)

[A] 2016

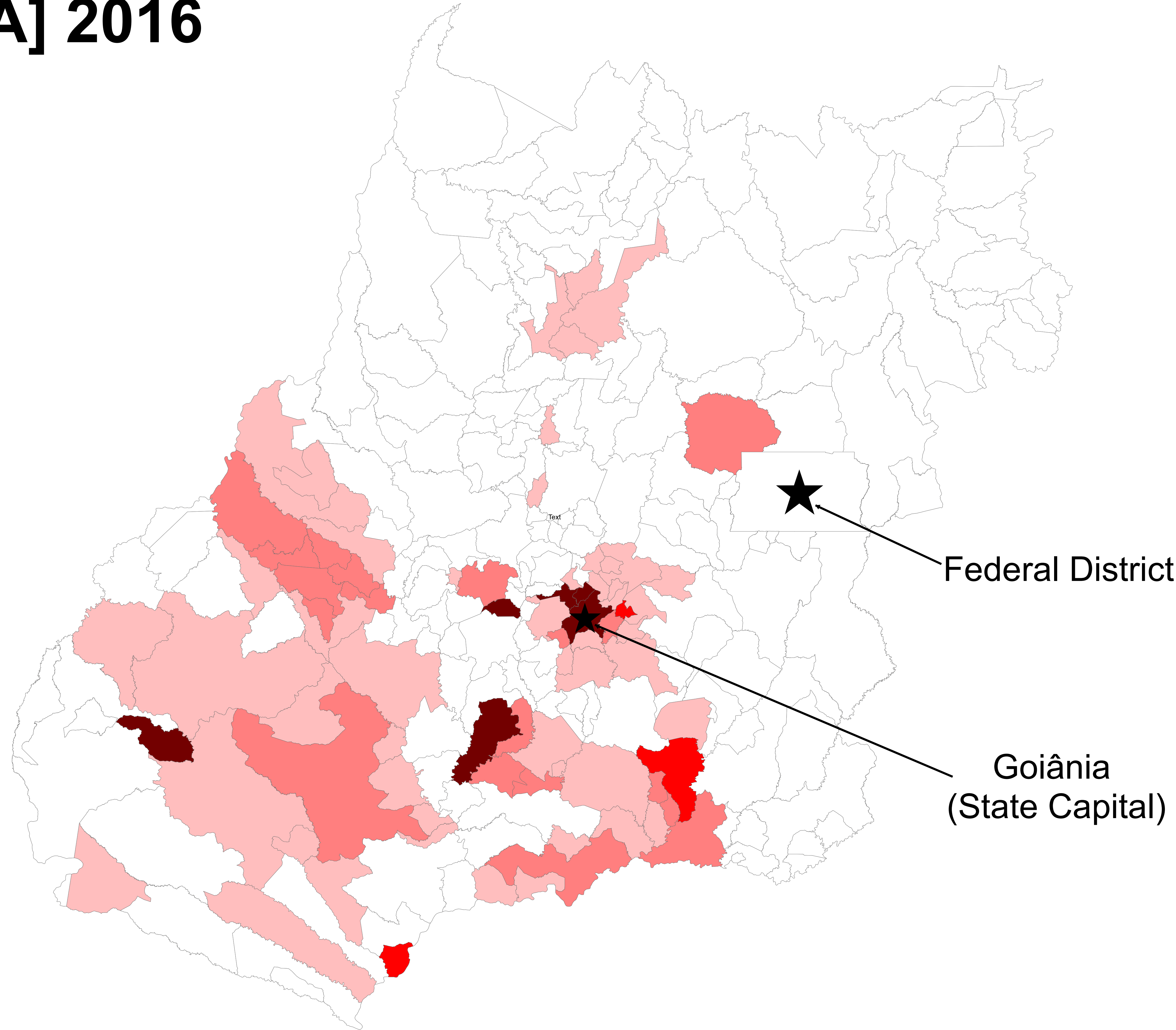

[B] 2017-2018

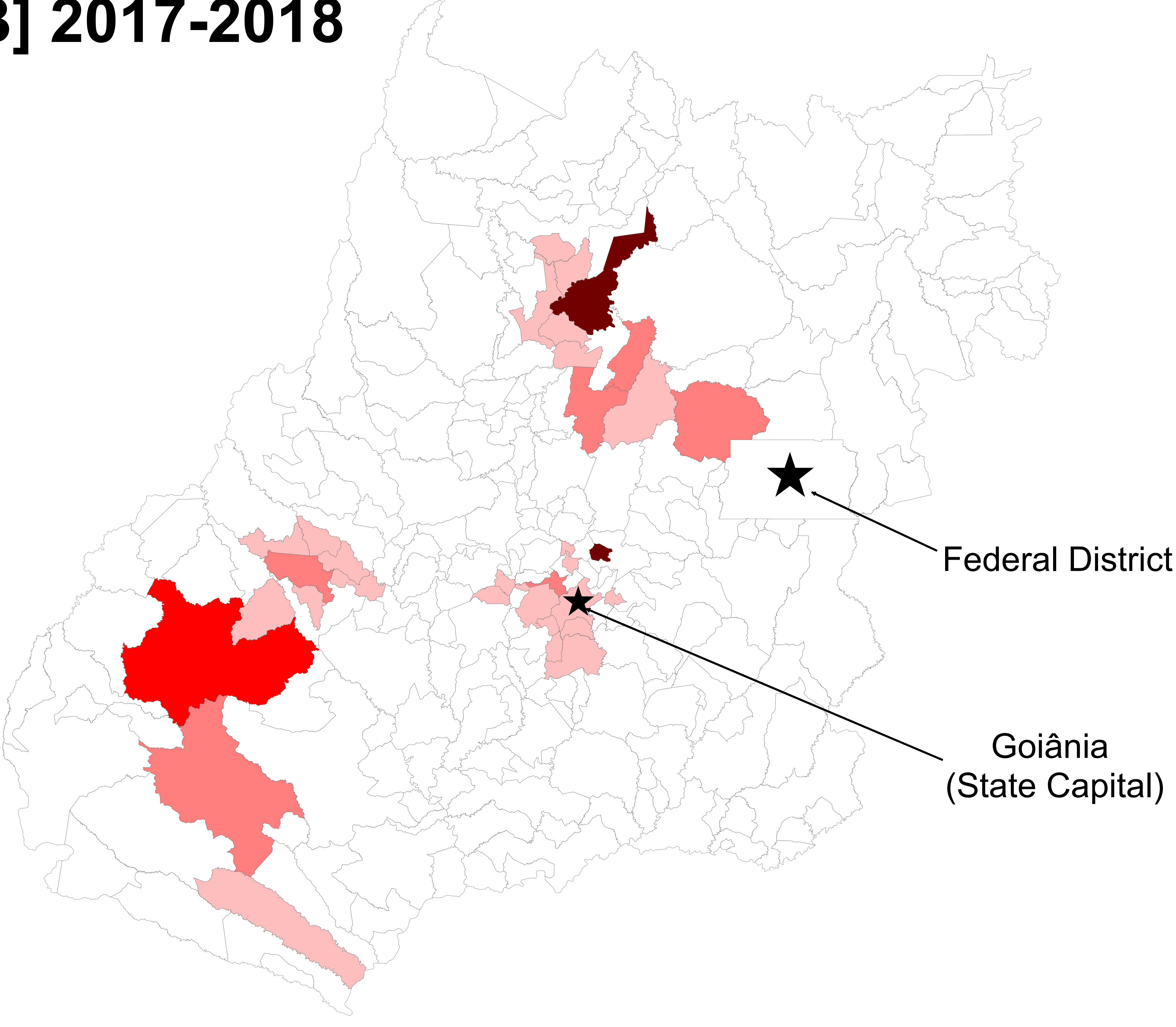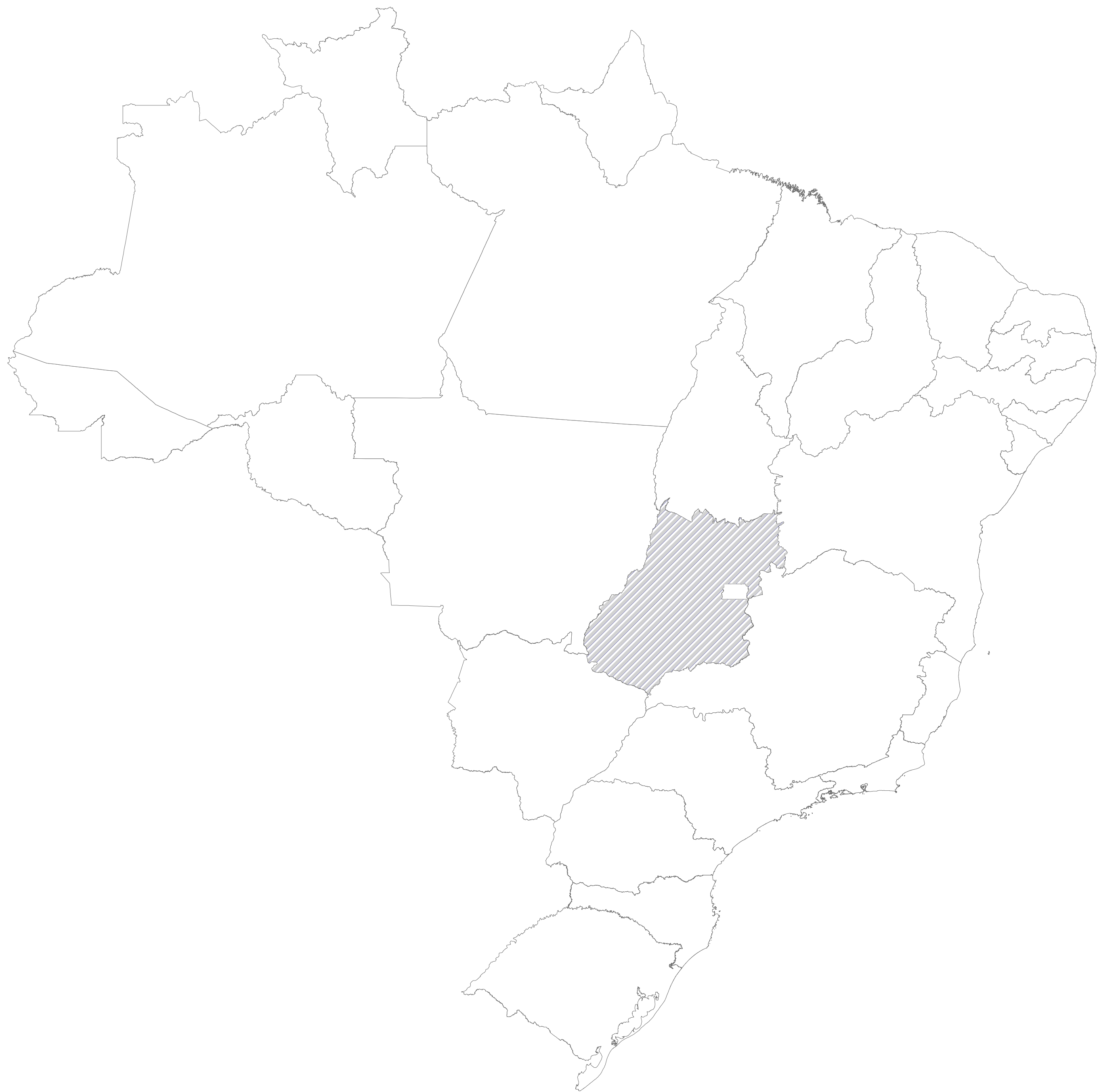

Brazil

Legend

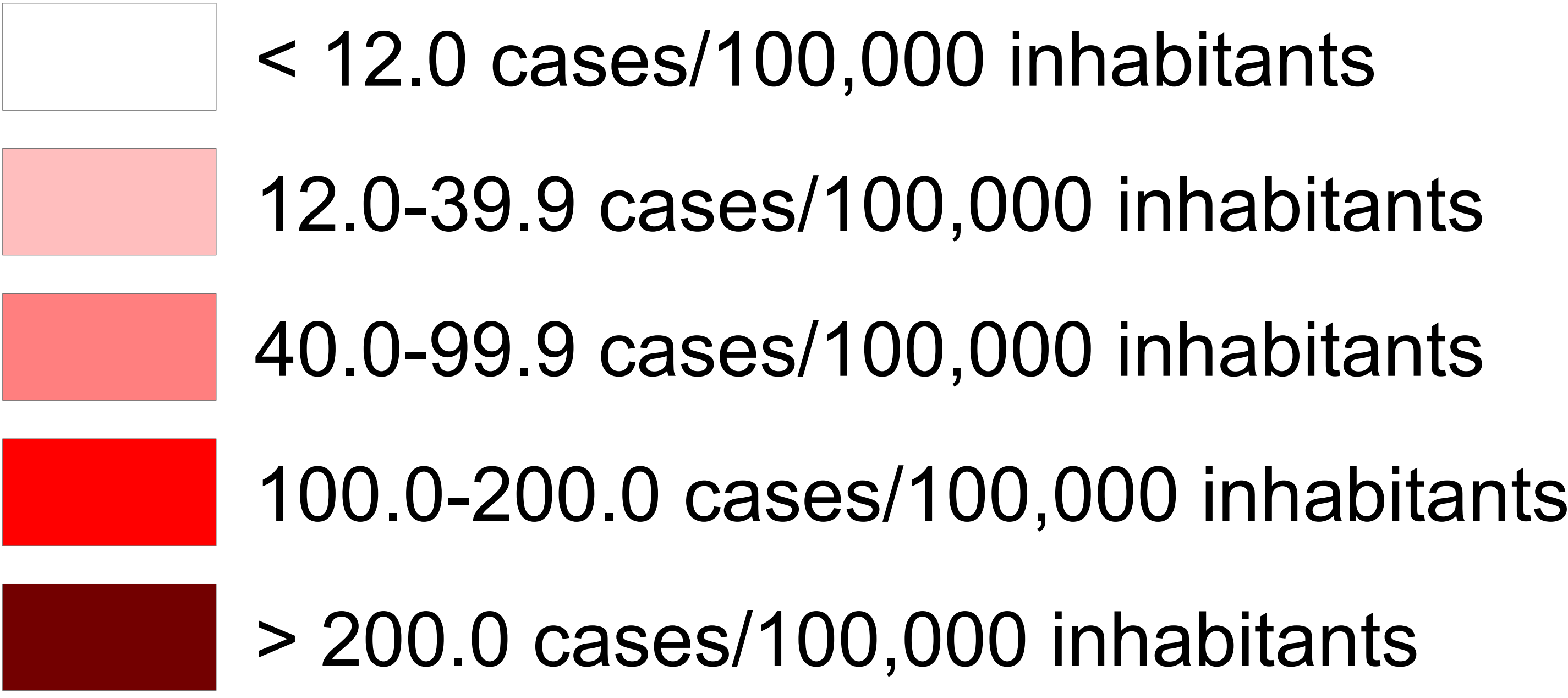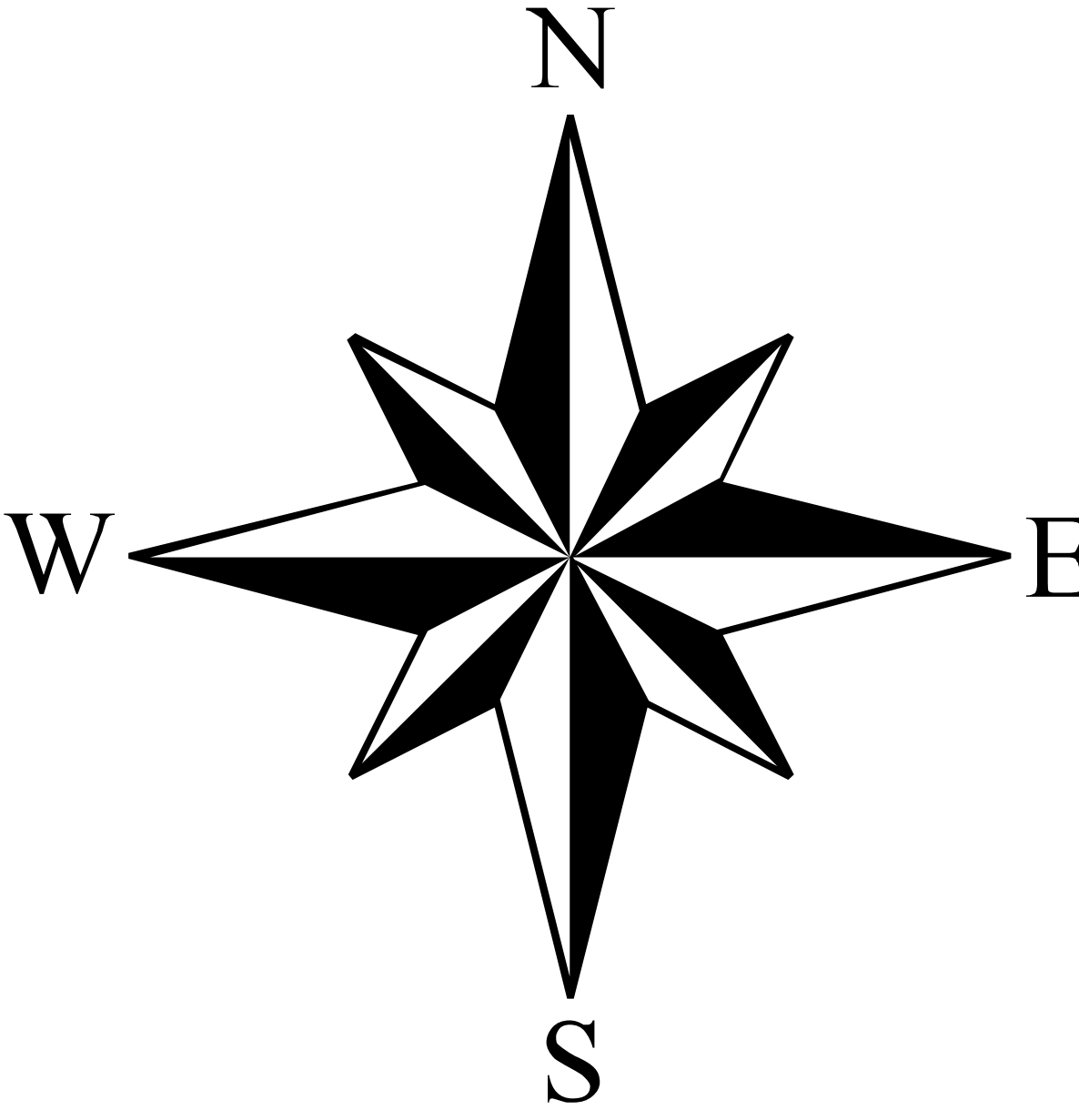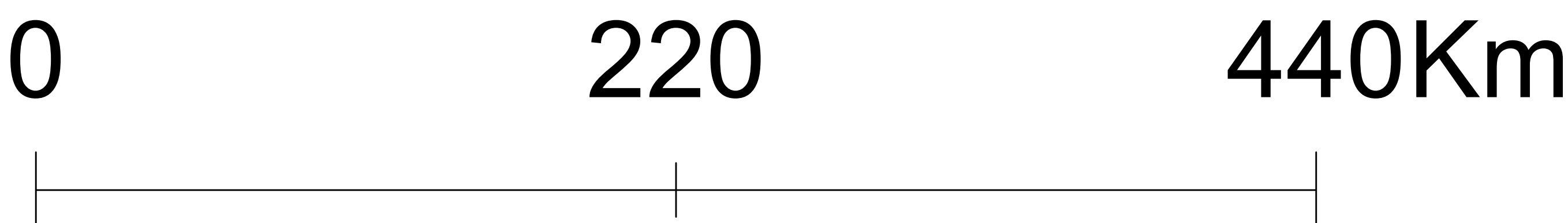

Supplement: Supplementary file 2 — Additional file 2: Figure S1. Descriptive spatial analysis of local empirical Bayesian rate of ZIKV infection in Goiás,Central-West region of Brazil, 2016-2018. Note: State-level scale. [file 12879_2021_6805_MOESM2_ESM.pdf]

[A] 2016

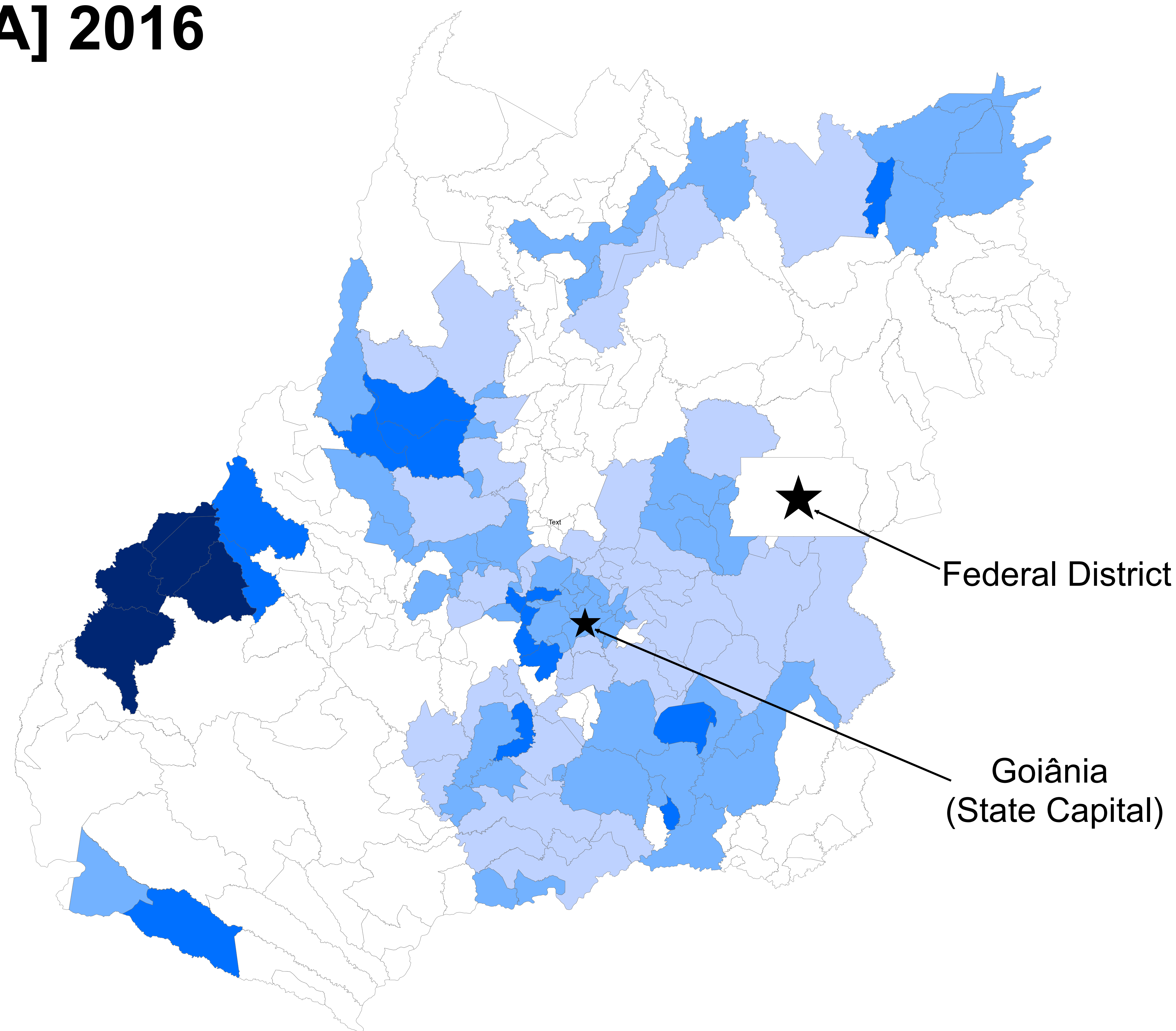

[B] 2017-2018

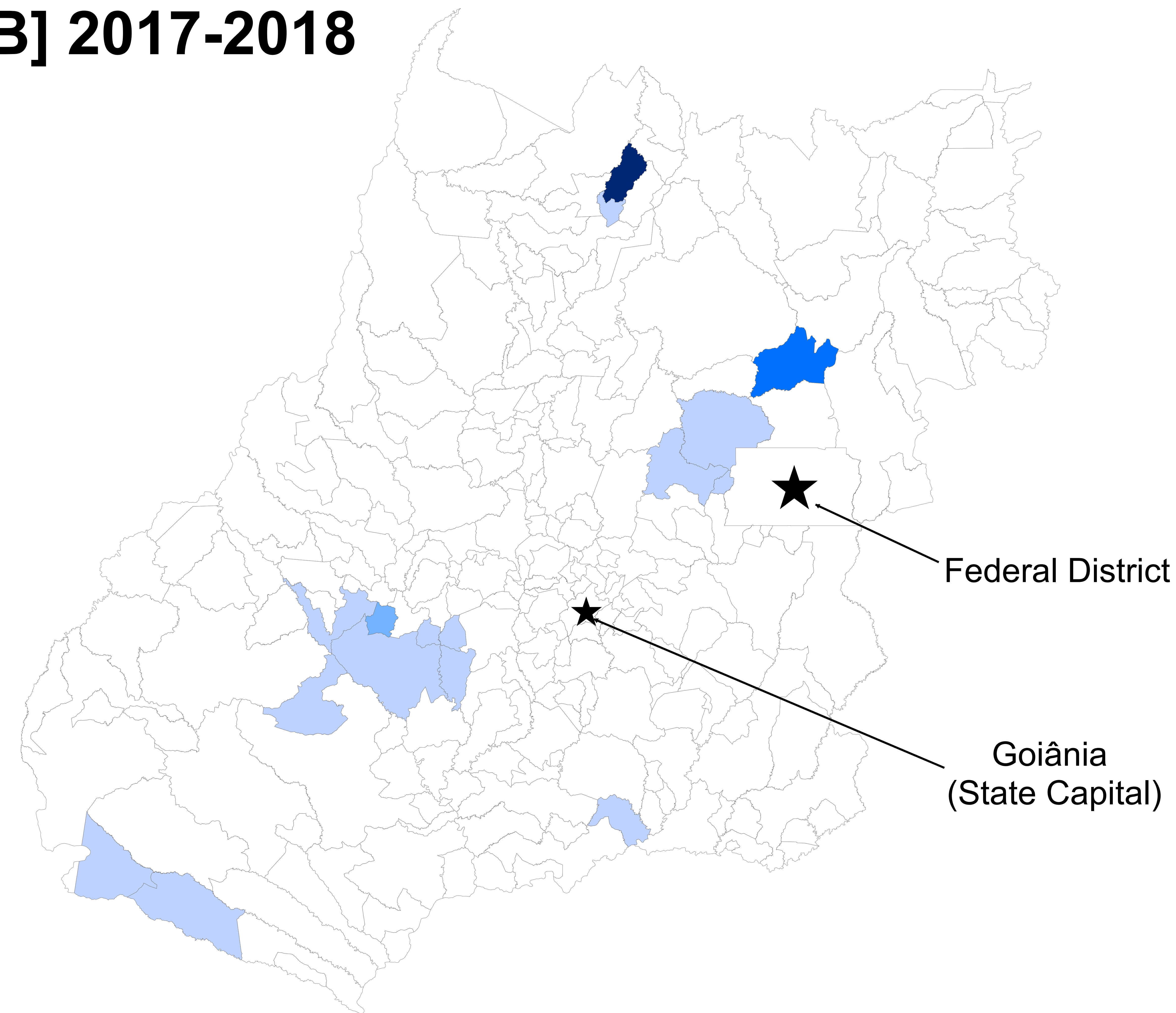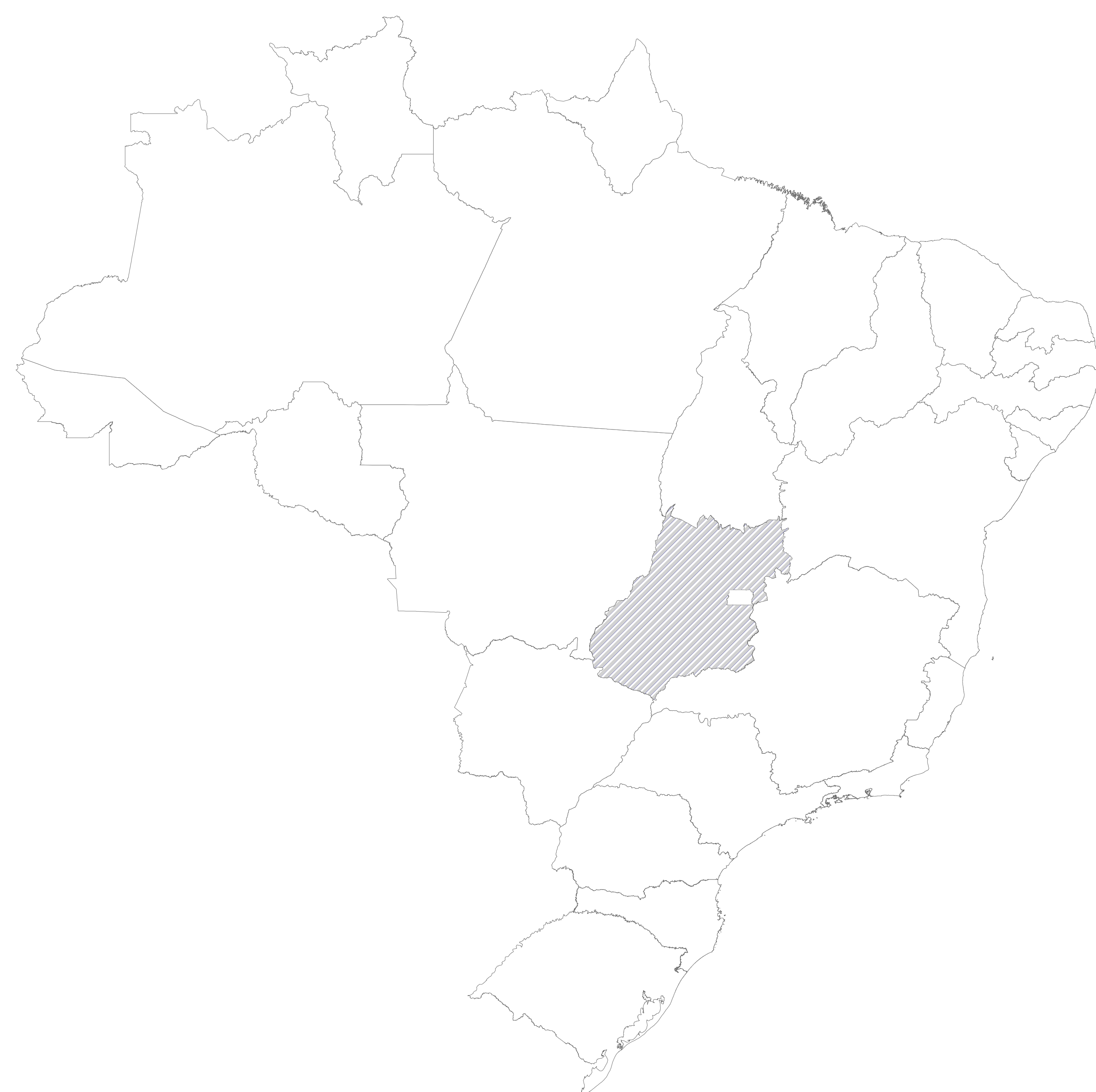

Brazil

**Legend**

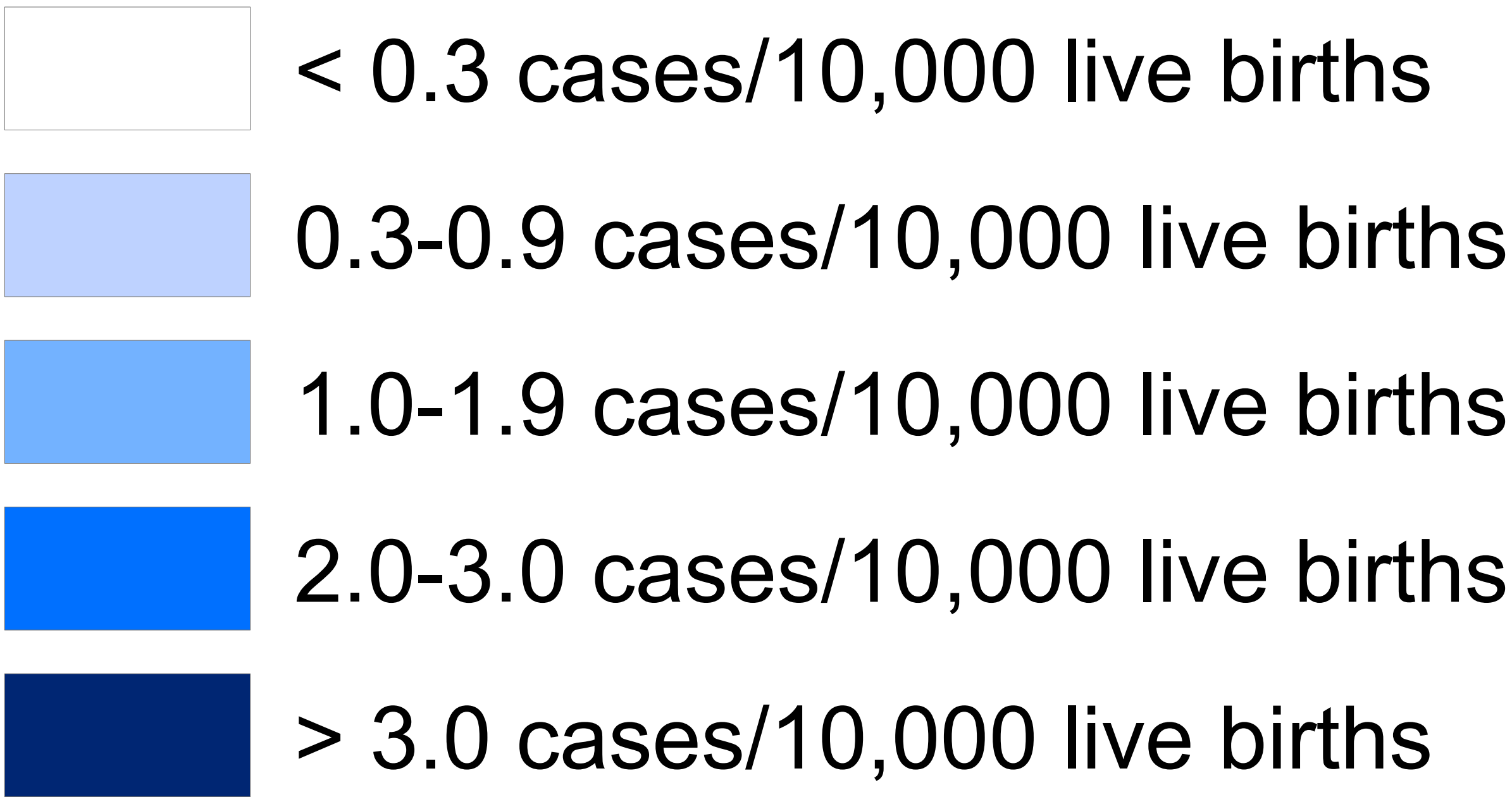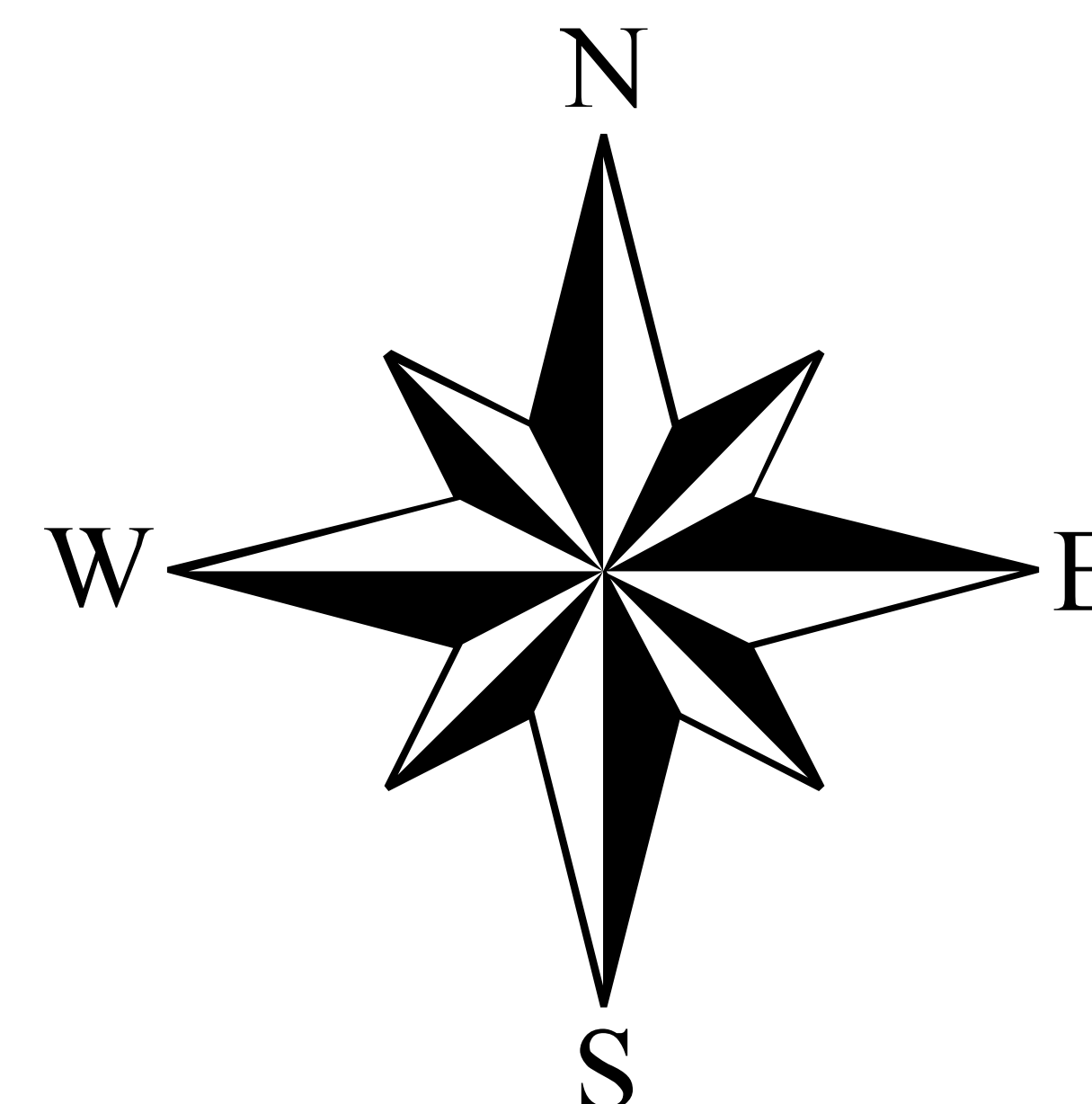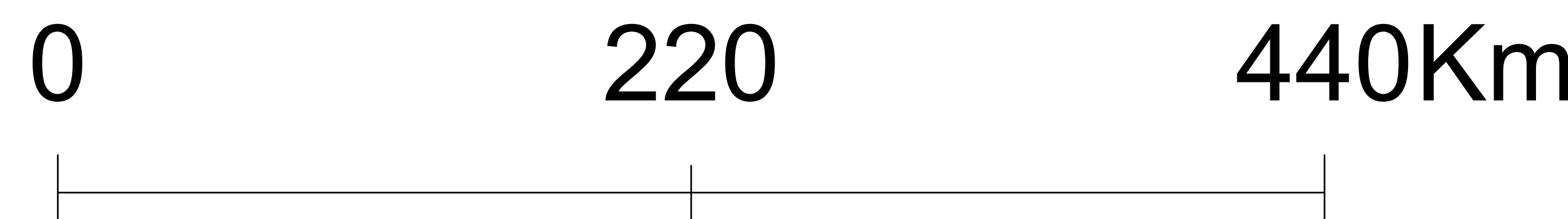

Supplement: Supplementary file 3 — Additional file 3: Figure S2. Descriptive spatial analysis of local empirical Bayesian rate of microcephaly and/or central nervous system alterations associated with congenital infection in Goiás, Central-West region of Brazil, 2016-2018. Note:State-level scale. [file 12879_2021_6805_MOESM3_ESM.pdf]

[A] 2016

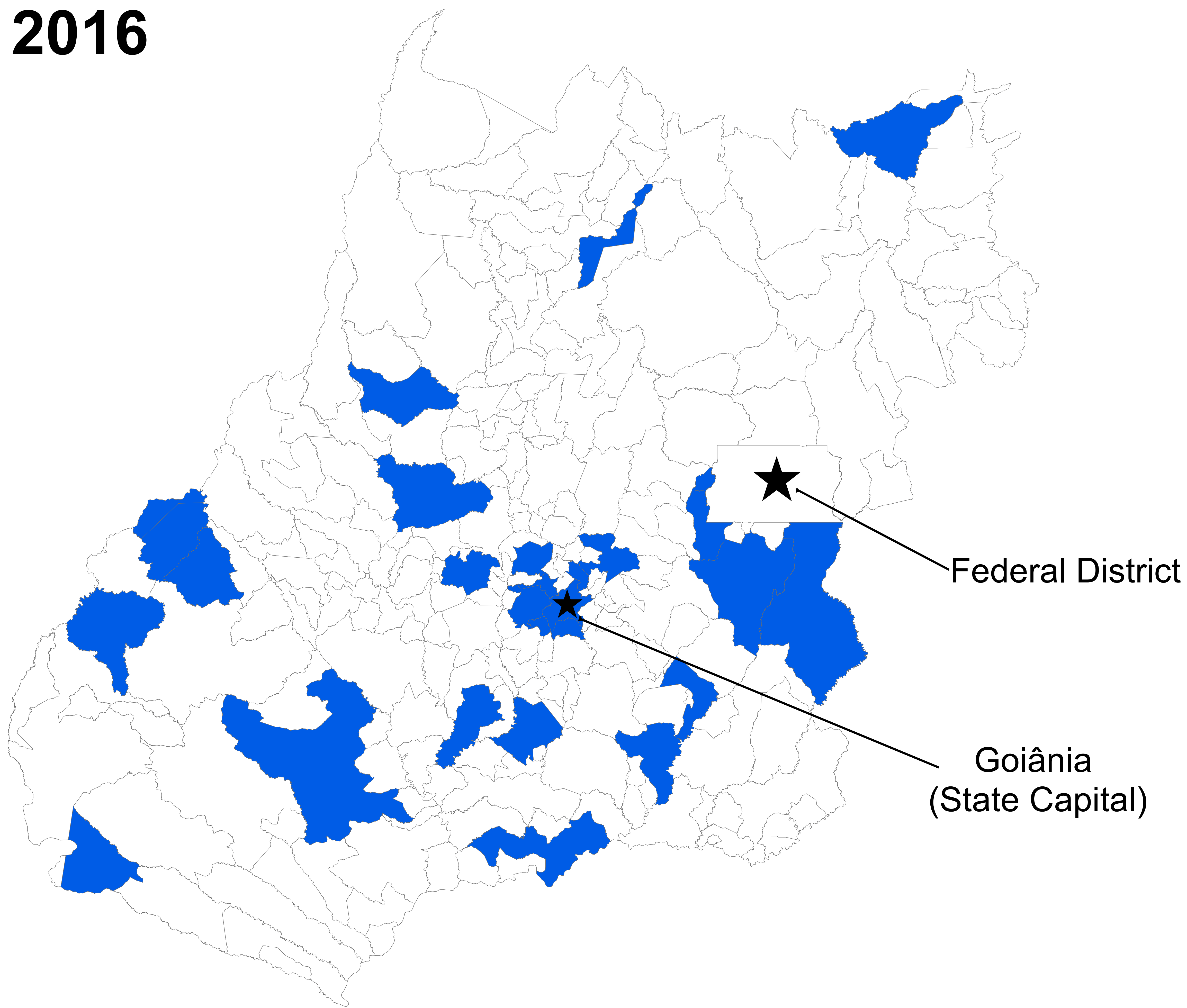

[B] 2017-2018

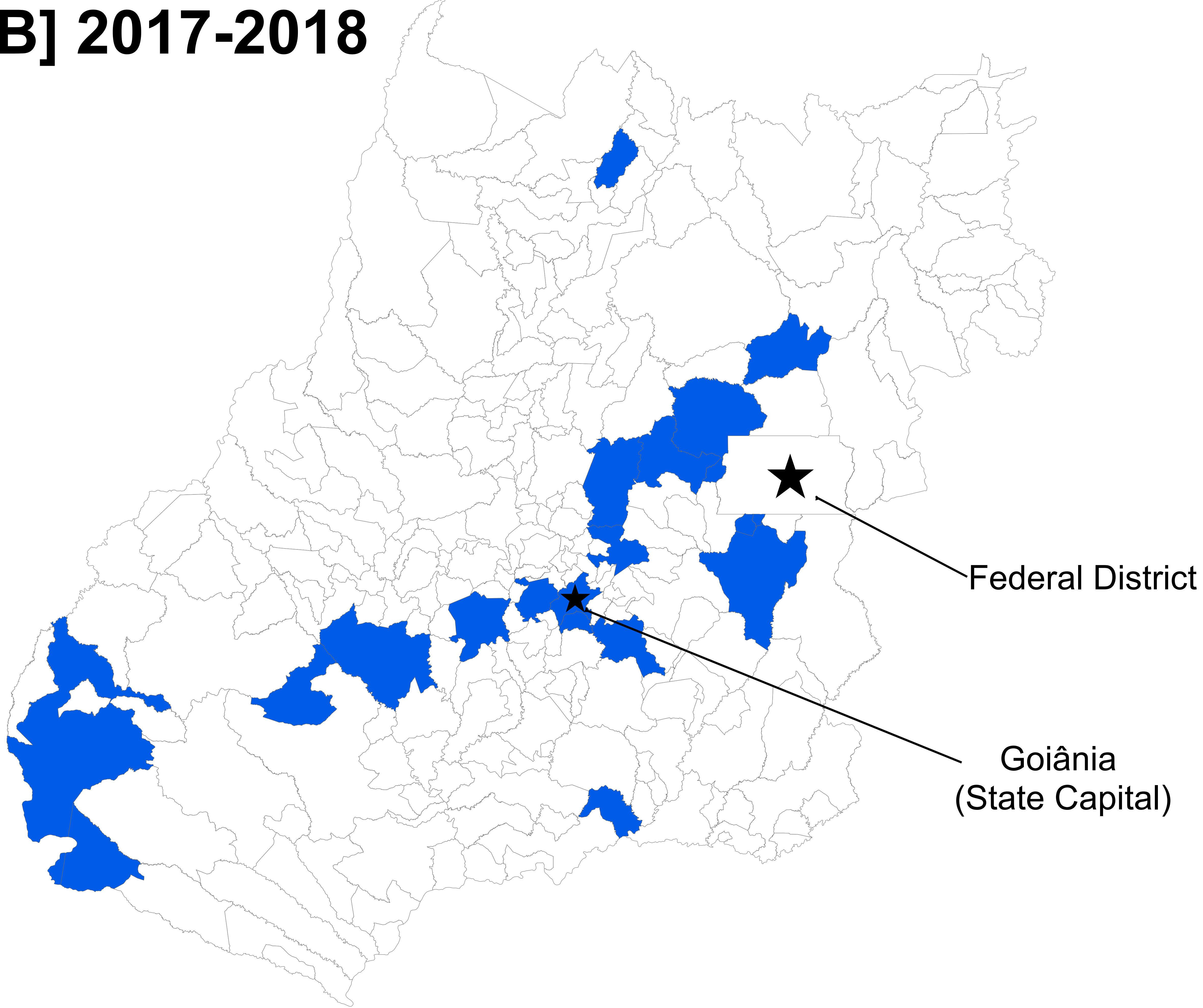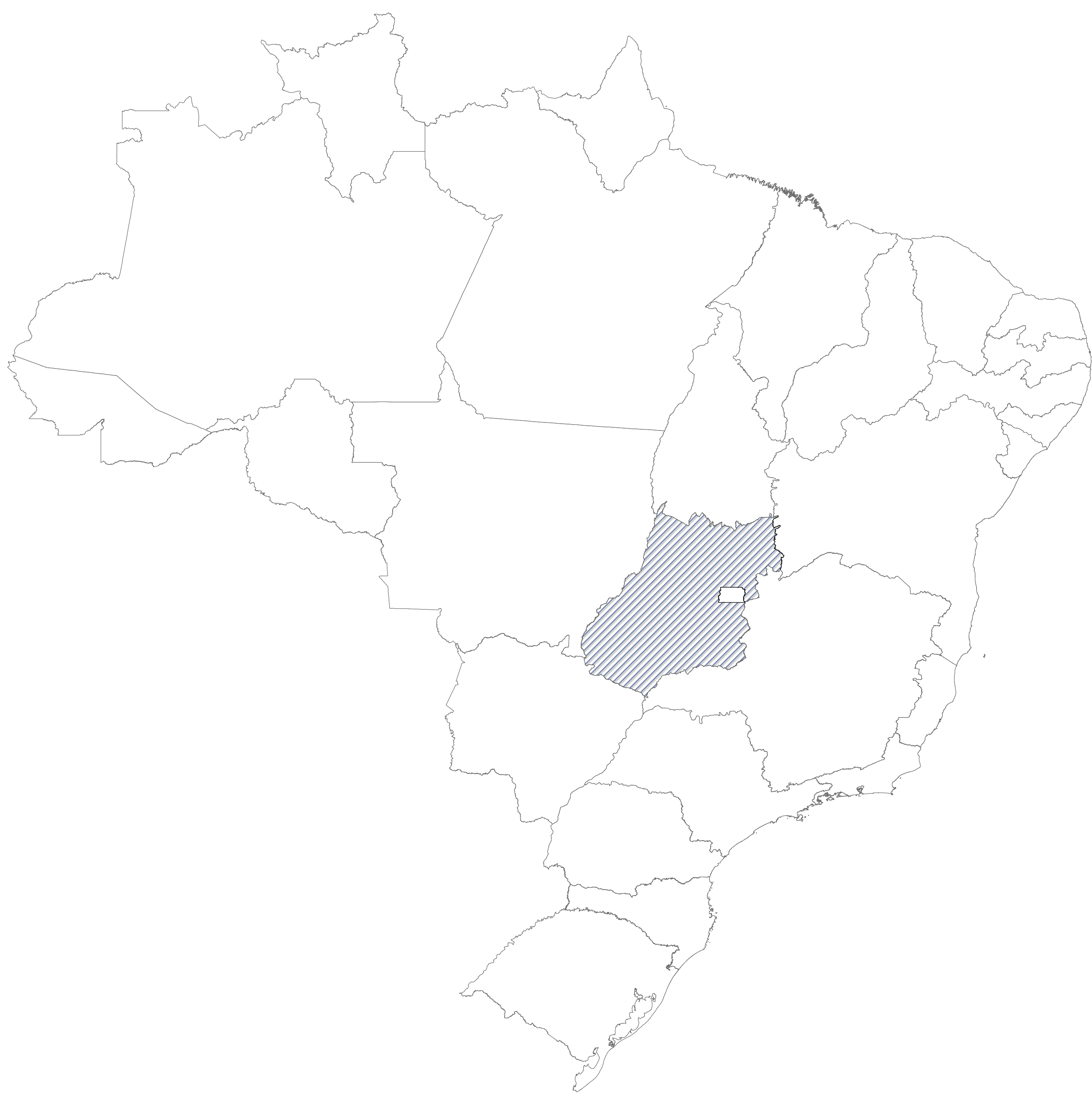

Brazil

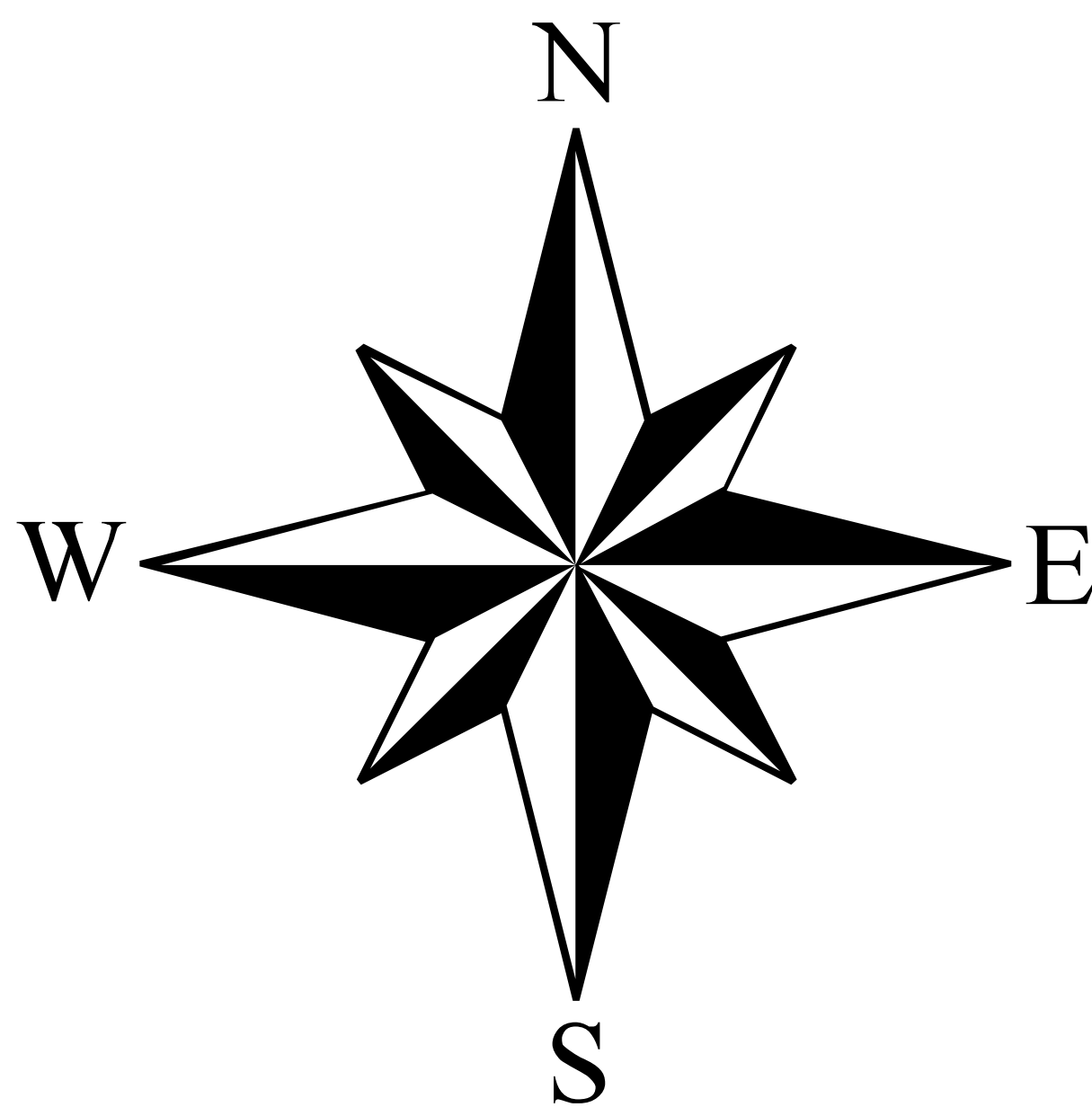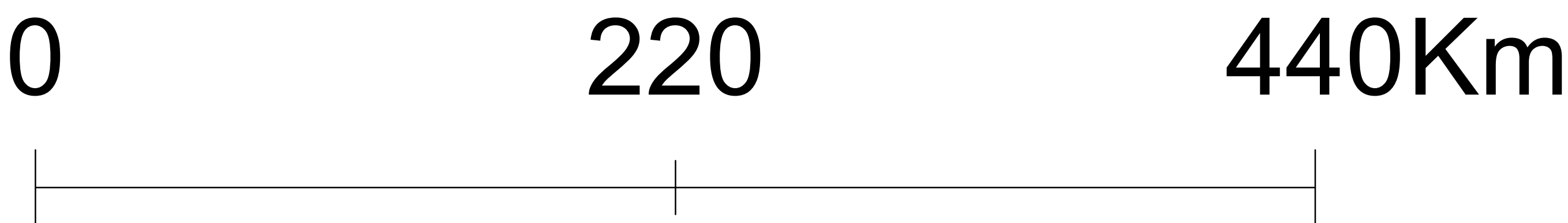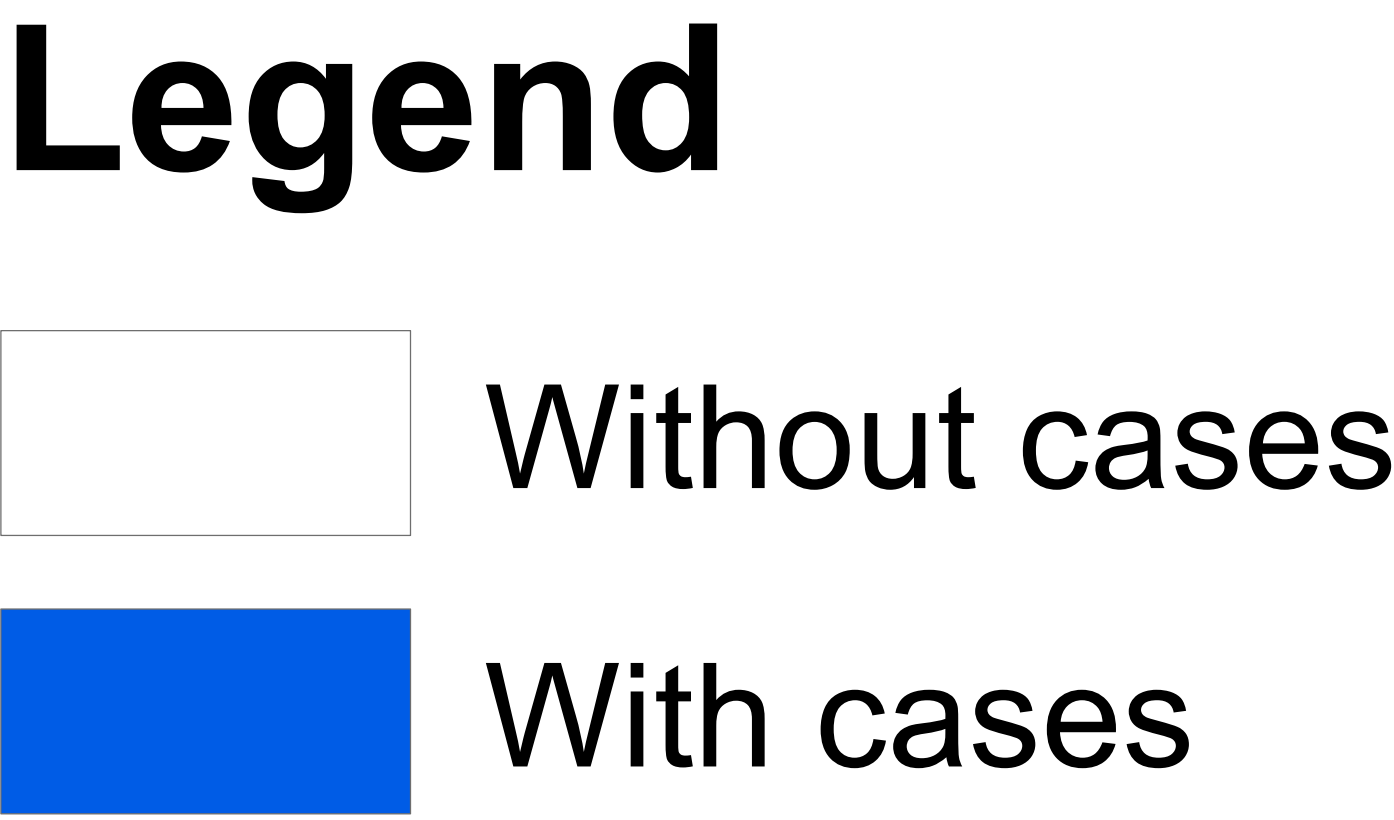

Supplement: Supplementary file 4 — Additional file 4: Figure S3. Municipalities with confirmed cases of microcephaly and/or central nervous system alterations associated with congenital infection in Goiás, Central-West region of Brazil,2016-2018. Note: State-level scale. [file 12879_2021_6805_MOESM4_ESM.pdf]
