# Supplementary material for: Zika virus infection and microcephaly: spatial analysis and socio-environmental determinants in a region of high Aedes aegypti infestation in the Central-West Region of Brazil
Source: BMC Infect Dis. 2021 Oct 27;21:1107. doi: 10.1186/s12879-021-06805-1 (PMC8549329; doi:10.1186/s12879-021-06805-1)
Supplement: Supplementary file 5 — Additional file 5: Table S1. Association between ecological determinants and the mean incidence rate of Zika virus infection inGoiás, Central-West region of Brazil, 2016-2018: bivariate analysis. [file 12879_2021_6805_MOESM5_ESM.docx]

**Table S1**. Association between ecological determinants and the mean incidence rate of Zika virus infection in Goiás, Central-West region of Brazil, 2016–2018: bivariate analysis.

| **Variables** |  | **Bivariate analysis** | | | | |
| --- | --- | --- | --- | --- | --- | --- |
|  | **cIRR (95%CI)** | | **β** | **EP** | **p*** |  |
| **Sociodemographic/economic** |  | |  |  |  |  |
| Gross domestic product per capita | 1.30 (1.28-1.33) | | 0.267 | 0.012 | <0.001 |  |
| Municipal human development index | 1.08 (1.06-1.09) | | 0.073 | 0.007 | <0.001 |  |
| Gini index | 2.78 (2.49-3.09) | | 1.020 | 0.151 | <0.001 |  |
| Population density | 0.90 (0.85-0.96) | | -0.099 | 0.027 | 0.001 |  |
| Gross domestic product per capita | 1.09 (1.08-1.10) | | 0.088 | 0.004 | <0.001 |  |
| **Health** |  | |  |  |  |  |
| Dengue fever incidence rate | 1.04 (1.03-1.05) | | 0.036 | 0.001 | <0.001 |  |
| Chikungunya incidence rate | 1.06 (0.98-1.14) | | 0.057 | 0.041 | 0.142 |  |
| Population coverage of community health agents | 0.86 (0.85-0.88) | | -0.149 | 0.008 | <0.001 |  |
| Population coverage of endemic disease control agents | 0.83 (0.81-0.84) | | -0.189 | 0.008 | <0.001 |  |
| **Environmental** |  | |  |  |  |  |
| Proportion of the population without access to sewage collection and treatment | 0.92 (0.91-0.93) | | -0.078 | 0.004 | <0.001 |  |
| Proportion of the population living in a household with piped water | 2.48 (2.26-2.70) | | 0.907 | 0.113 | <0.001 |  |
| Proportion of the population living in a garbage collection site | 4.37 (3.49-5.48) | | 1.474 | 0.503 | <0.001 |  |
| Average rainfall | 1.51 (1.42-1.61) | | 0.415 | 0.047 | <0.001 |  |
| Building infestation index for *Aedes* larvae | 2.22 (2.03-2.42) | | 0.797 | 0.099 | <0.001 |  |

β, regression coefficient; 95%CI, 95% confidence interval; cIRR, crude incidence rate ratio; *Wald Statistics.
